# Hyperedge approximation for stochastic processes on higher-order networks

**DOI:** 10.1073/pnas.2619078123

**Published:** 2026-08-05

**Authors:** Anzhi Sheng, Alex McAvoy, Ye Tian, Silun Zhang, Angela Fontan, Joshua B. Plotkin

**Affiliations:** ^a^ Department of Decision and Control Systems, KTH Royal Institute of Technology, Stockholm 10044, Sweden; ^b^ Department of Mathematics, KTH Royal Institute of Technology, Stockholm 10044, Sweden; ^c^ Department of Biology, University of Pennsylvania, Philadelphia, PA 19104; ^d^ School of Data Science and Society, University of North Carolina at Chapel Hill, Chapel Hill, NC 27599; ^e^ Department of Mathematics, University of North Carolina at Chapel Hill, Chapel Hill, NC 27599; ^f^ Center for Mathematical Biology, University of Pennsylvania, Philadelphia, PA 19014; ^g^ Meiji Institute for Advanced Study of Mathematical Sciences, Meiji University, Nakano, Tokyo 164-8525, Japan

**Keywords:** statistical physics, higher-order interactions, evolutionary game dynamics, complex contagion

## Abstract

Graphs provide a natural framework to describe processes shaped by pairwise interactions among agents. But many dynamical systems involve interactions within groups of three or more agents. Here, we develop the “ℓ-hyperedge approximation,” an analytical framework for stochastic processes on regular hypergraphs, in which each individual belongs to k groups of size ℓ. The framework accommodates both higher-order interactions that determine payoffs and higher-order processes for updating states in response to payoffs. For evolutionary games on hypergraphs, our analysis generalizes the classical b/c>k rule for cooperation to the ℓ-player donation game; and it yields critical benefit-to-cost ratios for the nonlinear ℓ-player public goods game, which remains bounded as the degree grows. Applied to neutral complex contagions, where inheritance of states occurs within hyperedges rather than along parent–offspring edges, the framework gives a closed-form fixation probability, showing how a single complexity parameter governs the spread of rare types. Coupling the two processes produces a unified stochastic model of payoff-biased complex contagions in structured populations. Together, these results extend pair approximation from graphs to hypergraphs, accommodating multiway interactions and group-level inheritance with no pairwise analog.

## Significance

How cooperation evolves in a population, and how new opinions take hold, depend on the structure of who interacts with whom. Most theories treat those interactions as pairwise—but many are not: Scientific collaborations, public goods contributions, and dinner-party debates often involve three or more participants at once. We develop a general theory of how behavior and opinions evolve when interactions happen in groups. The theory extends classical conditions for the evolution of cooperation to multiplayer games. It also shows how the spread of a rare type through group interactions is governed by the balance of novelty seekers and conformists. Our analysis provided a unified mathematical framework for studying multiparty group structure in both strategic interactions and behavioral updates.

Graphs provide a natural framework for describing the structure of complex systems (1–4). Each individual or agent is represented as a node, and interactions are captured by edges between pairs of nodes; the type of a node may change over time, as a result of these pairwise interactions. Pair approximation has become a standard technique for analyzing stochastic dynamics on graphs, with applications to the Ising model in physics (5), social dilemmas in evolutionary game theory (6, 7), and predator–prey dynamics in ecology (8). The method tracks the frequencies of edges of different types, while averaging over finer details of the network.

But many real-world interactions involve more than two individuals at once (9–13). Scientific collaborations, public-goods contributions, and group discussions all involve several agents simultaneously, with outcomes that pairwise decomposition cannot recover (14). Such higher-order interactions in a structured population are naturally described by hypergraphs, in which a hyperedge connects an arbitrary number of nodes. Two canonical group games illustrate this: the public goods game, in which each member’s contribution benefits all other players (15–17), and the multiplayer donation game, in which a single cooperator confers benefits to the entire group.

Higher-order interactions can also shape how states are updated over time, independent of the gameplay. On pairwise graphs, states propagate from parent to offspring along an edge—simple contagion (18–25). On hypergraphs, updates occur at the group level, and the rate at which a node adopts a type may depend nonlinearly on the number of neighbors already holding that type (so-called “complex” contagion) (26–32). In opinion dynamics, for example, the probability that an individual adopts a particular opinion may increase synergistically, or saturate, with the number of peers already holding it. Such nonlinearities in the update process, even in the absence of fitness effects, fall outside the scope of pair approximation and of existing coalescent-based methods (33–36).

Here we develop the ℓ-hyperedge approximation, a framework for analyzing stochastic processes on regular hypergraphs in which each node belongs to k hyperedges each of order ℓ. The framework accommodates higher-order interactions both in the payoff-generating phase—evolutionary games on hypergraphs—and in the state-updating phase—complex contagion. We benchmark the approximation against the fixation probability of a rare strategy or opinion (37), and we extend it to a combined model in which payoff-biased selection and complex contagion act simultaneously.

For evolutionary games on hypergraphs, where higher-order interactions shape only the payoff-generating phase, analysis of the ℓ-player donation game yields a generalization of the classical pairwise result (6): Cooperation is favored whenever the benefit-to-cost ratio exceeds the average degree, b/c>k. For the nonlinear ℓ-player public goods game, we compute the critical benefit-to-cost ratio for cooperation as a function of the degree k and a benefit factor δ that encodes synergy or antagonism among contributions. For neutral complex contagion, where higher-order interactions shape only the inheritance process, we derive a closed-form expression for the fixation probability and show how the complexity parameter governs the rate at which a rare opinion spreads. Coupling the two processes yields a unified stochastic model of payoff-biased complex contagion, and we show how conformity and novelty tendencies shift the threshold for the spread of cooperation.

## Model

### Stochastic Processes on Hypergraphs.

**Fig. 1.**
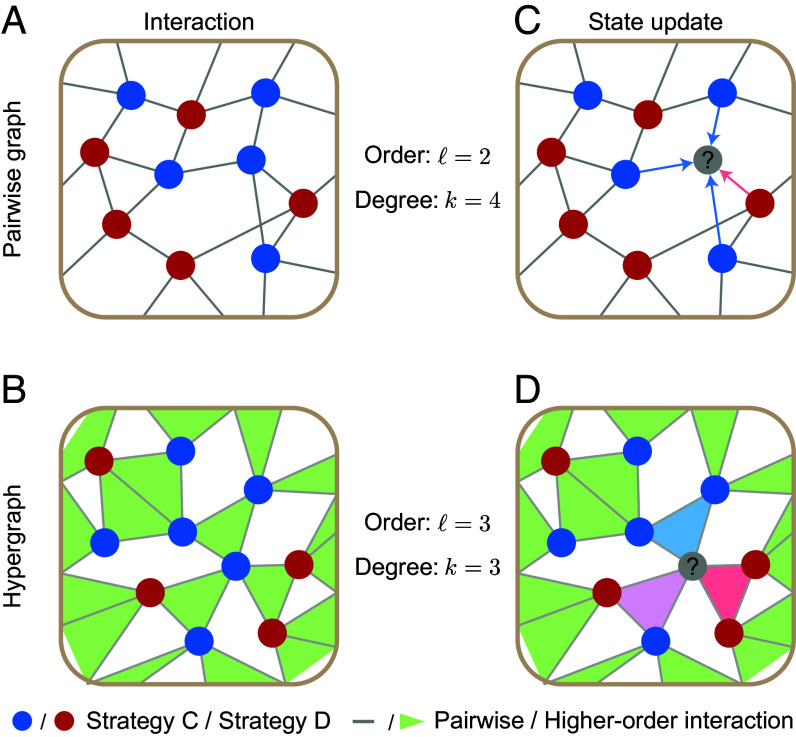
Strategy evolution on graphs and hypergraphs. The population structure is represented by a regular network of order ℓ; we consider ℓ=2 (pairwise graph) and ℓ=3 (third-order hypergraph). Interactions (gameplay) are represented by gray lines and green triangles, respectively. (*A* and *B*), Each individual occupies a node and adopts either strategy C (blue circles) or strategy D (red circles). Each individual then interacts with their neighbors in either two- or three-player games, and receives accumulated payoffs. (*C* and *D*), At each time step, a random individual i (labeled “?”) is selected uniformly to update their strategy. The probability of adopting strategy C is positively related to: i) the number of (hyper)edges containing $n_{C}$ neighbors in state C and $n_{D}$ neighbors in state D, and ii) the rate of adopting C in such edges, denoted by $F_{C}\left(n_{C},n_{D}\right)$ if i is in state D, or by $G_{C}\left(n_{C},n_{D}\right)$ if i is in state C (Eqs. **1** and **2**). Corresponding expressions provide the chance of adopting strategy D. On a pairwise graph (*C*), strategy updating reduces to standard death-birth dynamics (6), where the selected individual i simply imitates the strategy of a neighbor with a probability that depends on their payoffs.

We describe a broad class of stochastic processes on hypergraphs, including those with frequency-dependent selection. Consider a population of N individuals whose structure is represented by a regular hypergraph of order ℓ: Each node denotes an individual, and each hyperedge captures an interaction within a group of ℓ individuals. Fig. 1 *A* and *B* illustrate two cases we analyze in detail: ℓ=2 (pairwise graph) and ℓ=3 (third-order hypergraph).

Each individual holds one of two strategies, C or D, and participates in k order-ℓ interactions. The hyperedges attached to a focal individual are classified into 2ℓ categories of the form $\left(X,n_{C},n_{D}\right)$, where X∈{C,D} is the strategy of the focal individual, and $n_{C}+n_{D}=\ell -1$ is the composition of the ℓ−1 remaining neighbors in the hyperedge. Let $k_{n_{C},n_{D}}$ denote the number of hyperedges of composition $\left(X,n_{C},n_{D}\right)$ attached to the focal individual, so that $\sum_{n_{C}+n_{D}=\ell -1}k_{n_{C},n_{D}}=k$.

After all interactions occur, a random individual i is selected to update their strategy. If i previously adopted D, the probability that i updates their strategy to C is

[1] $$\begin{array}{c} p_{D\to C}=\frac{\sum_{n_{C}+n_{D}=\ell -1}k_{n_{C},n_{D}}F_{C}\left(n_{C},n_{D};\;\theta \right)}{\sum_{n_{C}+n_{D}=\ell -1}k_{n_{C},n_{D}}\left(\begin{array}{c} F_{C}\left(n_{C},n_{D};\;\theta \right)\\ +F_{D}\left(n_{C},n_{D};\;\theta \right) \end{array}\right)}, \end{array}$$

where $F_{C}\left(n_{C},n_{D};\;\theta \right)$ and $F_{D}\left(n_{C},n_{D};\;\theta \right)$ denote the weights associated to the chances of i adopting C and D in a hyperedge of type $\left(D,n_{C},n_{D}\right)$, respectively. Likewise, if i previously adopted C, the probability of remaining at C is

[2] $$\begin{array}{c} p_{C\to C}=\frac{\sum_{n_{C}+n_{D}=\ell -1}k_{n_{C},n_{D}}G_{C}\left(n_{C},n_{D};\;\theta \right)}{\sum_{n_{C}+n_{D}=\ell -1}k_{n_{C},n_{D}}\left(\begin{array}{c} G_{C}\left(n_{C},n_{D};\;\theta \right)\\ +G_{D}\left(n_{C},n_{D};\;\theta \right) \end{array}\right)}, \end{array}$$

where $G_{C}\left(n_{C},n_{D};\;\theta \right)$ and $G_{D}\left(n_{C},n_{D};\;\theta \right)$ denote the weights associated to the chances of i adopting C or D in a hyperedge of type $\left(C,n_{C},n_{D}\right)$.

The parameter θ≥0 represents the intensity of selection (38). We focus on the regime of weak selection, i.e θ≪1, a standard assumption in evolutionary biology and sociophysics (6, 33, 39–42). Moreover, we assume that, in the absence of selection (θ=0), the strategy dynamics are fair in expectation in the sense that no strategy is favored and the global frequency of cooperation $p_{C}$ evolves as a martingale.

On a pairwise graph (ℓ=2), this update reduces to standard death-birth dynamics (6): The focal individual copies the strategy of a single neighbor (Fig. 1*C*), perhaps biased by their relative payoffs. On a hypergraph (ℓ≥3), the focal individual is influenced simultaneously by all ℓ−1 coplayers in the selected hyperedge, and the adoption rate may depend nonlinearly on their composition (Fig. 1*D*). The hypergraph update is not reducible to an iteration of pairwise imitations, nor is there a single “parent” from which a focal individual inherits their strategy.

### The ℓ-Hyperedge Approximation and Fixation Probability.

To analyze the stochastic process above, we extend the classical pair approximation for regular graphs to an ℓ-hyperedge approximation. Let $p_{C}$ denote the global frequency of strategy C, and let $q_{\left(n_{C},n_{D}\right)|C}$ and $q_{\left(n_{C},n_{D}\right)|D}$ denote the conditional probabilities that, given a focal individual adopting C or D, a randomly chosen hyperedge containing the focal individual has composition $\left(n_{C},n_{D}\right)$ among its ℓ−1 remaining positions. Together, $p_{C}$ and the conditional frequencies determine the state of the system at the hyperedge level.

Under weak selection, the hyperedge frequencies $q_{\left(n_{C},n_{D}\right)|C}$ and $q_{\left(n_{C},n_{D}\right)|D}$ equilibrate on a faster timescale than the global frequency $p_{C}$. At quasi-equilibrium, each conditional frequency can be expressed as a polynomial in $p_{C}$ (Eq. **14** in *Materials and Methods* and *SI Appendix*, section 1.3). A central identity governs these quasi-equilibrium relations:

[3] $$\begin{array}{c} \sum_{n_{C}+n_{D}=\ell -1}n_{C}\left(q_{\left(n_{C},n_{D}\right)|C}-q_{\left(n_{C},n_{D}\right)|D}\right)=\frac{1}{k-1}, \end{array}$$

which indicates that a C-individual has, on average, one more C-neighbor than a D-individual across its k−1 other hyperedges. Eq. **3** generalizes the positive-assortment identity of Ohtsuki et al. (6) to hypergraphs of arbitrary order.

Our primary object of interest is the fixation probability $\rho_{C}$: The probability that a single C mutant, introduced at a uniformly random node in an otherwise all-D population, eventually takes over the whole population. Because the process has no mutation, the population eventually reaches one of two absorbing states (all-C or all-D); $\rho_{C}$ quantifies how readily C invades.

To derive the fixation probability $\rho_{C}$, we track the dynamics of $p_{C}$, $q_{\left(n_{C},n_{D}\right)|C}$, and $q_{\left(n_{C},n_{D}\right)|D}$ throughout the process using the ℓ-hyperedge approximation (*SI Appendix*, section 1). Since $q_{\left(n_{C},n_{D}\right)|C}$ and $q_{\left(n_{C},n_{D}\right)|D}$ are polynomial functions of $p_{C}$, it is sufficient to track the expected change and the variance in $p_{C}$ over a single time step, denoted by $E(\Delta p_{C})$ and $\text{Var}(\Delta p_{C})$, respectively. Using the polynomial closure together with a diffusion approximation (Eq. **24** in *Materials and Methods* and *SI Appendix*, section 2), we obtain

[4] $$\begin{array}{cc} \rho_{C} & =\frac{1}{N}+\frac{\theta }{N}\sum_{m=0}^{\ell -1}\frac{1}{\left(m+1\right)\left(m+2\right)}{\bar{\tau }}^{\left(m\right)}+O\left(\theta^{2}\right), \end{array}$$

for sufficiently large N, where the coefficients ${\bar{\tau }}^{\left(m\right)}$ are the coefficients of $2E\left(\Delta p_{C}\right)/\;\text{Var}\left(\Delta p_{C}\right)$ when expanded as a polynomial in $p_{C}$ (see *SI Appendix*, section 2 for more details).

## Results

We apply Eq. **4** to three classes of dynamics on hypergraphs: evolutionary games, where higher-order interactions determine payoffs; neutral complex contagion, where higher-order interactions determine state updates; and payoff-biased complex contagion, where both phases are affected.

### Evolutionary Games on Hypergraphs.

In the context of evolutionary game dynamics, strategy C represents cooperation and strategy D represents defection. Each ℓth-order interaction is a game with ℓ concurrent players: A focal cooperator receives payoff $a_{n_{C}}$, and a focal defector receives payoff $b_{n_{C}}$, when $n_{C}$ of the ℓ−1 coplayers choose cooperation.

After all games have been played, each individual accumulates a payoff from their k order-ℓ interactions. Then, a random individual i is selected to update their strategy by imitating one of their neighbors. Following the death-birth updating rule (6), the probability that i imitates a given neighbor j is proportional to j’s fitness and to the number of interactions in which i and j both participate. Concretely,

[5] $$\begin{array}{c} \begin{array}{cc} F_{C}\left(n_{C},n_{D};\;\theta \right) & =n_{C}\exp\left(\theta u_{C}\left(n_{C},n_{D}\right)\right),\\ F_{D}\left(n_{C},n_{D};\;\theta \right) & =n_{D}\exp\left(\theta u_{D}\left(n_{C},n_{D}\right)\right),\\ G_{C}\left(n_{C},n_{D};\;\theta \right) & =n_{C}\exp\left(\theta v_{C}\left(n_{C},n_{D}\right)\right),\\ G_{D}\left(n_{C},n_{D};\;\theta \right) & =n_{D}\exp\left(\theta v_{D}\left(n_{C},n_{D}\right)\right), \end{array} \end{array}$$

where $u_{C}\left(n_{C},n_{D}\right)$ and $u_{D}\left(n_{C},n_{D}\right)$ represent the accumulated payoffs to a cooperator neighbor and a defector neighbor in the hyperedge $\left(D,n_{C},n_{D}\right)$, respectively, and $v_{C}\left(n_{C},n_{D}\right)$ and $v_{D}\left(n_{C},n_{D}\right)$ represent the corresponding payoffs to a cooperator and defector neighbor in the hyperedge $\left(C,n_{C},n_{D}\right)$. Payoffs determine individuals’ fitness f= exp(θu). Substituting Eq. **5** into Eq. **4** gives the fixation probability of cooperation on hypergraphs of order ℓ (Eq. **30**).

We focus on the cases ℓ=2 (standard graphs) and ℓ=3 (order-three hypergraphs). We are interested in understanding when selection favors the evolution of cooperation, namely under what conditions the fixation probability of cooperation under weak selection exceeds that under neutral drift ($\rho_{C}>1/N$). For ℓ=2, this condition becomes

[6] $$\begin{array}{c} \begin{array}{c} \left(2k^{2}-2k-1\right)a_{0}+\left(k^{2}+2k+1\right)a_{1}\\ >\left(2k^{2}+k-1\right)b_{0}+\left(k^{2}-k+1\right)b_{1}, \end{array} \end{array}$$

which recovers the well-known result derived by the pair approximation (6). When ℓ=3, the condition becomes

[7] $$\begin{array}{c} \begin{array}{cc} & \left(12k^{3}-16k^{2}+3k+3\right)a_{0}+\left(8k^{3}-6k-6\right)a_{1}\\ & +\left(4k^{3}+4k^{2}+3k+3\right)a_{2}>\left(12k^{3}-9k+3\right)b_{0}\\ & +\left(8k^{3}-8k^{2}+6k-6\right)b_{1}+\left(4k^{3}-4k^{2}+3k+3\right)b_{2}. \end{array} \end{array}$$

A parallel condition for the relative success of cooperation over defection, $\rho_{C}>\rho_{D}$, is derived in *Materials and Methods* (Eq. **33**). This condition is identical to that for the absolute success ($\rho_{C}>1/N$, Eq. **7**) when the game is additive (i.e. linear), in which case a multiplayer game on higher-order interactions can be reduced to a collective of pairwise games on pairwise edges. However, when the game exhibits nonlinearity, higher-order interactions come into play, and the two conditions differ.

#### Donation games.

Our first application of this general result is to determine what (if any) multiplayer payoff structure preserves the classical b/c>k rule for the spread of cooperation. Eq. **7** reduces to b/c>k under the assignment $a_{0}=-c$, $a_{1}=-c+b$, $a_{2}=-c+2b$, $b_{0}=0$, $b_{1}=b$, and $b_{2}=2b$. More generally, we prove (Eq. **32** in *Materials and Methods*) that on hypergraphs of arbitrary order ℓ, cooperation is favored whenever b/c>k, under the payoff scheme $a_{n_{C}}=-c+n_{C}b$ and $b_{n_{C}}=n_{C}b$. We refer to this payoff structure as the ℓ-player donation game: A cooperator pays cost c and provides benefit b to each of its ℓ−1 coplayers, while a defector pays nothing and provides nothing. The total benefit provided by a single cooperator is B=(ℓ−1)b, so larger groups require a proportionally larger total benefit to sustain cooperation.

The intuition behind the b/c>k rule on hypergraphs follows directly from the fundamental identity built into the ℓ-hyperedge approximation (Eq. **3**). From Eq. **3**, a cooperator has on average one additional cooperative neighbor, across its k−1 other hyperedges, than a defector. This assortment produces an expected payoff difference of b−kc between a cooperator and a defector. Cooperation is favored exactly when this difference is positive, which yields b/c>k (*SI Appendix*, Fig. S1).

The ℓ-player donation game is additive, and so its analysis reduces to a sequence of pairwise donation games [by rescaling cost to c/(ℓ−1) and degree to k(ℓ−1)]. The theory of traditional pair approximation would therefore suffice to analyze the evolution of cooperation in this higher-order donation game; and so the result above is best seen as a consistency check that our method of higher-order analysis produces the expected, generalized result for higher-order donation games. Furthermore, in this case, the condition for the absolute success of cooperation ($\rho_{C}>1/N$) is equivalent to that for the relative success ($\rho_{C}>\rho_{D}$).

#### Public goods games.

Our framework for studying higher-order interactions extends to nonlinear multiplayer games, which cannot be studied by reduction to pairwise games. As an illustrative example we compute the threshold for cooperation in the classic ℓ-player public goods game, on a regular hypergraph.

In the public goods game, a cooperator pays a cost c to contribute to a shared benefit that is distributed equally among all ℓ players (regardless of strategy), while a defector pays nothing. The total benefit scales nonlinearly with the number of cooperators (36, 43):

[8] $$\begin{array}{c} \begin{array}{cc} a_{n_{C}} & =\frac{b(1-\delta^{n_{C}+1})}{\ell (1-\delta )}-c,\\ b_{n_{C}} & =\frac{b(1-\delta^{n_{C}})}{\ell (1-\delta )}, \end{array} \end{array}$$

where the benefit factor δ controls the nonlinearity: δ=1 is the linear case, δ>1 encodes synergy (each additional cooperator produces more benefit), and δ<1 encodes antagonism in the production of public benefits.

Before turning to the hypergraph, consider the classical, well-mixed case. In a single one-shot game played among ℓ players with no spatial structure, a cooperator’s marginal return on its own contribution is $b\delta^{n_{C}}/\ell$ when there are $n_{C}-1$ other cooperators in the group. For the linear public goods game (δ=1) this marginal return is independent of $n_{C}$, and the game admits a single, sharp Nash threshold ${\left(b/c\right)}^{*}=\ell$: Below it defection is the dominant strategy; above it cooperation is. For δ≠1, the well-mixed game has two thresholds rather than one: ${\left(b/c\right)}^{*}=\ell$, where the all-defector state ceases to be Nash, and ${\left(b/c\right)}^{*}=\ell /\delta^{\ell -1}$, where the all-cooperator state becomes Nash. In every case the thresholds grow with group size. The question for the hypergraph is whether spatial structure—and the positive assortment of cooperators it induces—can shift these baselines.

On a large regular hypergraph, substituting the public-goods payoffs into Eqs. **6** and **7** yields a closed-form expression for the critical benefit-to-cost ratio to favor cooperation:

[9] $$\begin{array}{c} {\left(\frac{b}{c}\right)}^{*}=\left\{\begin{array}{cc} & \frac{6k^{2}}{\left(k+1)(k(\delta +2)+\delta -1\right)},\;\ell =2,\\ & \frac{36k^{2}\left(2k-1\right)}{\left(k+1\right)\left(\begin{array}{c} \delta^{2}\left(4k^{2}+3\right)+\delta \left(8k^{2}-6\right)+3{\left(2k-1\right)}^{2} \end{array}\right)},\;\ell =3. \end{array}\right. \end{array}$$

**Fig. 2.**
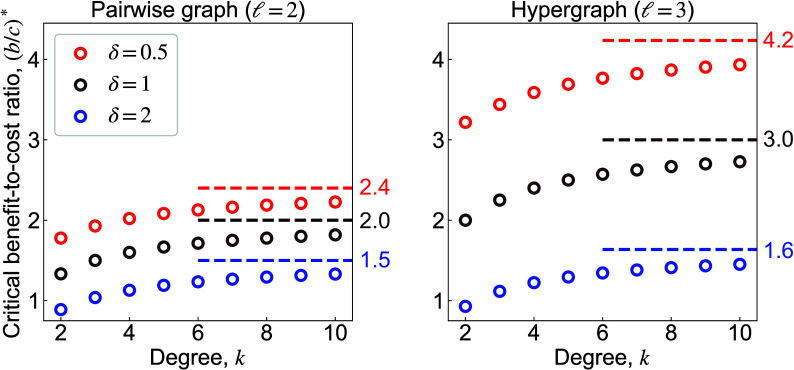
Threshold for cooperation in public goods games. Theoretical predictions for the critical benefit-to-cost ratio, ${\left(b/c\right)}^{*}$, required for cooperation to be favored in the public goods game, for hypergraphs of different orders ℓ and degrees k. For fixed benefit factor δ, the critical ratio ${\left(b/c\right)}^{*}$ increases monotonically with degree k and approaches a constant as k→∞, corresponding to the well-mixed limit. For any given degree k, the critical ratio is larger for ℓ=3 than for ℓ=2, indicating that public goods games played in larger groups generate a stronger social dilemma.

Fig. 2 plots the critical ratio ${\left(b/c\right)}^{*}$ as a function of the degree k, for both ℓ=2 and ℓ=3 and several values of the benefit factor δ. Note that these critical ratios are derived under the condition for the absolute success ($\rho_{C}>1/N$), different from those based on the relative success ($\rho_{C}>\rho_{D}$) when the game is nonlinear (δ≠1).

Two qualitative features stand out. First, for any fixed δ and k, the threshold for ℓ=3 exceeds the threshold for ℓ=2: The social dilemma intensifies in larger groups, consistent with the donation-game intuition that a single cooperator’s contribution must be shared among more coplayers. Second, in contrast to the donation game, where the threshold ${\left(b/c\right)}^{*}=k$ diverges with the degree, the public goods threshold remains bounded as k→∞, converging to 6/(δ+2) for ℓ=2 and to $18/(\delta^{2}+2\delta +3)$ for ℓ=3. This result reflects the fact that the donation game presents a stronger social dilemma than the public goods game. In the public goods game a cooperator always retains a share of their own contribution, whereas in the donation game a cooperator gains nothing unless another cooperator is present in their hyperedge.

The spatial cooperation threshold (Eq. **9**) in the well-mixed limit has a precise relationship to the well-mixed Nash conditions. For ℓ=3, $\lim_{k\to \infty }{\left(b/c\right)}^{*}=18/\left(\delta^{2}+2\delta +3\right)$. This value coincides with the well-mixed Nash boundaries b/c=ℓ and $b/c=\ell /\delta^{\ell -1}$ only at δ=1, where all three thresholds collapse to ${\left(b/c\right)}^{*}=\ell =3$. For δ≠1, the limiting threshold lies strictly inside the well-mixed Nash interval and matches neither endpoint. The reason is that the marginal return $b\delta^{n_{C}}/\ell$ depends on the local composition $n_{C}$ whenever δ≠1: The well-mixed Nash analysis evaluates this return at extreme compositions ($n_{C}=0$ for all-defector stability, $n_{C}=\ell -1$ for all-cooperator stability), whereas the death-birth dynamics on a hypergraph averages it over the quasi-equilibrium distribution of compositions experienced by a focal cooperator versus a focal defector. Spatial structure thus selects a single, sharp cooperation threshold from within the range that the well-mixed Nash analysis can only bracket.

Finite k further lowers the cooperation threshold relative to the well-mixed (k→∞) limit. For δ=1, the closed forms collapse to the simple expression ${\left(b/c\right)}^{*}=\ell k/\left(k+1\right)$ for both ℓ=2 and ℓ=3, which lies strictly below the well-mixed Nash threshold ℓ. The reduction is largest when k is small: A sparse hypergraph imposes the strongest positive assortment among cooperators, and therefore the greatest discount on the cost of contributing to the public good. The same monotonicity persists for δ≠1 (Fig. 2): For any fixed δ, ${\left(b/c\right)}^{*}$ decreases as k decreases, so spatial structure always makes cooperation easier than the well-mixed limit alone would predict.

**Fig. 3.**
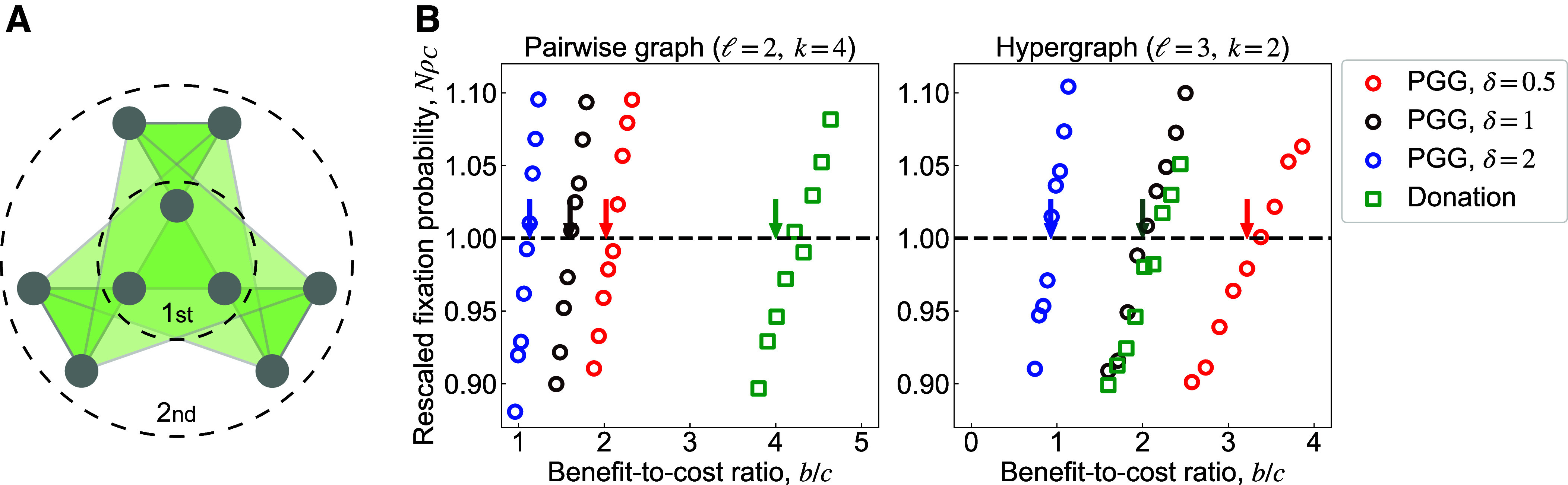
Monte Carlo simulations confirm theoretical predictions for evolutionary games on hypergraphs. (*A*), A homogeneous population structure organized into m layers. The nth layer contains $3\cdot 2^{n-1}$ nodes, so the total population size is $N=3(2^{m}-1)$. Every pair of nodes in the nth layer is connected to a single node in the (n−1)th layer. The degree is k=4 for a graph with pairwise interactions (ℓ=2), and k=2 for a structure with three-way interactions (ℓ=3). (*B*), Rescaled fixation probability $N\rho_{C}$ as a function of the benefit-to-cost ratio b/c, for the donation game and the public goods game. Circles denote averages across $2\times 10^{6}$ independent Monte Carlo simulations; arrows indicate theoretical predictions from the ℓ-hyperedge approximation. Parameters: m=5 (N=95), c=1, and θ=0.01.

We validate these analytical predictions against Monte Carlo simulations on a regular hypergraph (Fig. 3*A*) in which nodes are organized into m layers, the nth layer contains $3\cdot 2^{n-1}$ nodes, and the total population size is $N=3(2^{m}-1)$. The degree is k=4 for ℓ=2 and k=2 for ℓ=3. Fig. 3*B* shows that Monte Carlo estimates of $N\rho_{C}$ agree with the ℓ-hyperedge approximation, for both the donation game and the public goods game, across a range of b/c values. The approximation also remains accurate on random regular hypergraphs generated by the configuration model and even on heterogeneous hypergraphs whose degree varies across nodes (*SI Appendix*, Fig. S2).

### Complex Contagions on Hypergraphs.

#### Complex contagion without payoff bias.

In the analysis of evolutionary games above, higher-order interactions enter only through payoffs, while state updates still follow a simple contagion process based on pairwise comparisons: That is, the rates in Eq. **5** are linear in $n_{C}$ and $n_{D}$. We now turn to complex contagion, where the likelihood that an individual adopts a particular strategy has a nonlinear dependence on how many individuals adopt that strategy within the hyperedge—so that higher-order structure shapes the inheritance process, not just the payoffs.

Following the literature on complex contagion (30, 31), we first consider the neutral case in which each strategy has the same fitness, so that dynamics arise from the contagion process alone. In this context, strategies C and D can be interpreted as two competing opinions, but neither opinion has an intrinsic advantage over the other. Specifically, we posit the following rates for strategic updating,

[10] $$\begin{array}{c} \begin{array}{cc} F_{C}\left(n_{C},n_{D};\;\theta \right) & =G_{C}\left(n_{C},n_{D};\;\theta \right)=n_{C}^{1+\lambda \theta },\\ F_{D}\left(n_{C},n_{D};\;\theta \right) & =G_{D}\left(n_{C},n_{D};\;\theta \right)=n_{D}^{1+\lambda \theta }, \end{array} \end{array}$$

which are controlled by a complexity parameter λ. When λ=0, the dynamics reduce to simple contagion. For λ>0 and λ<0, the dynamics follow complex contagion, corresponding to conformity-seeking and novelty-seeking behavior, respectively.

Complex contagion is an inherently group-level phenomenon. The inheritance of states can no longer be represented by parent-to-offspring maps, but rather inheritance arises from the entire composition of states in the group of size ℓ. On a pairwise graph, the rates in Eq. **10** are either 0 or 1 regardless of λ, so the complexity parameter has no effect on the dynamics; complex contagion requires ℓ≥3. For the same reason, existing analytical approaches based on parent-to-offspring inheritance and coalescent theory (33, 34, 44, 45) do not apply to complex processes on hypergraphs. For complex contagion, inheritance is mediated by the hyperedge as a whole rather than by a single parent, and the standard coalescent constructions break down.

To quantify the effect of complexity on the spread of a rare type, or opinion, we consider the invasion strength, the leading-order effect of selection on the fixation probability of one type: $\partial_{\theta }\rho_{C}{|}_{\theta =0}$. In particular, we study how the invasion strength changes with the average degree k and the complexity parameter λ. For ℓ=3, the invasion strength is

**Fig. 4.**
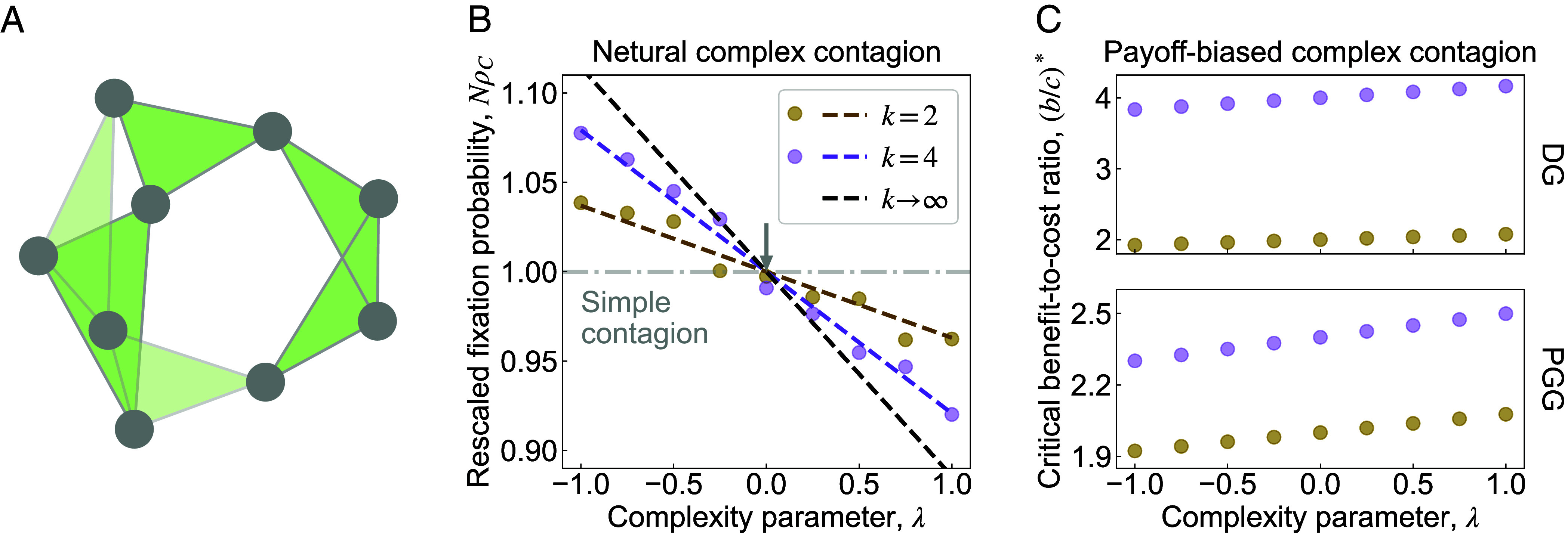
Complex contagion on hypergraphs. We consider complex contagion dynamics on third-order random regular hypergraphs generated by the configuration model (*SI Appendix*, Algorithm 1). (*A*), An illustrative example of a random regular hypergraph of size N=9 and degree k=2. (*B*), Rescaled fixation probability, $N\rho_{C}$, under neutral complex contagion, where the two strategies have the same fitness. Dots show results from $2\times 10^{6}$ independent Monte Carlo simulations, and dashed lines show the theoretical predictions. For fixed degree k, the fixation probability decreases monotonically with the complexity parameter λ: Conformity-seeking dynamics (λ>0) suppress the spread of a rare type, whereas novelty-seeking dynamics (λ<0) promote it. For fixed λ, the deviation from neutrality becomes stronger as k increases and approaches a limiting value as k→∞. (*C*), Effect of complex contagion on cooperation when contagion dynamics are combined with the donation game (DG) or the public goods game (PGG). Increasing λ raises the critical ratio ${\left(b/c\right)}^{*}$, indicating that conformity hinders, whereas novelty promotes, the evolution of cooperation. Parameters: c=1, θ=0.01, and N=99 for simulations.

[11] $$\begin{array}{c} \partial_{\theta }\rho_{C}{|}_{\theta =0}=-\frac{\lambda \left(\ln\;2\right)\left(2k-3\right)}{6\left(2k-1\right)} \end{array}$$

in the large population limit (Eq. **36** in *Materials and Methods*). This expression shows that the sign of the invasion strength is determined entirely by λ: Novelty-seeking dynamics (λ<0) promote the spread of a rare type, whereas conformity-seeking dynamics (λ>0) suppress it. Moreover, for fixed λ, the magnitude of the invasion strength increases monotonically with degree k. Thus the effect of complex contagion is weakest on sparsely connected hypergraphs and becomes stronger as the population approaches the well-mixed limit. When the average degree approaches that limit, k→∞, the invasion strength converges to −λln2/6. Monte Carlo simulations on random regular hypergraphs (Fig. 4 *A* and *B*) confirm both the quantitative predictions in Eq. **11** and these qualitative trends.

These results admit a simple interpretation. When λ>0, the update rule is conformity-seeking: The effective influence of a strategy grows more than linearly with the number of individuals already holding it in the hyperedge. A rare type therefore faces an additional disadvantage, because it typically appears in groups where it is underrepresented, and so its fixation probability is reduced. When λ<0, by contrast, the update rule is novelty-seeking: The influence of a strategy grows sublinearly with local abundance, which softens the disadvantage of being rare and increases the chance that a rare type spreads. In this sense, a positive complexity parameter acts like an additional discounting effect on rare opinions, whereas a negative complexity parameter acts like a synergistic effect that favors their initial growth.

#### Complex contagion with payoff-biased reproduction.

We now combine the two mechanisms studied above: payoff-biased selection from evolutionary game dynamics and nonlinear state updating from complex contagion. In this setting, higher-order interactions affect both phases of the process. They determine payoffs through the game played within each hyperedge, and they also determine how strongly the local composition of the hyperedge biases subsequent strategy updating. Thus the same group structure shapes both fitness and inheritance.

To model this combined process, we let adoption rates depend multiplicatively on payoff and on the complexity of contagion. The resulting rates are

[12] $$\begin{array}{c} \begin{array}{cc} F_{C}\left(n_{C},n_{D};\;\theta \right) & =n_{C}^{1+\lambda \theta }\exp\left(\theta u_{C}\left(n_{C},n_{D}\right)\right),\\ F_{D}\left(n_{C},n_{D};\;\theta \right) & =n_{D}^{1+\lambda \theta }\exp\left(\theta u_{D}\left(n_{C},n_{D}\right)\right),\\ G_{C}\left(n_{C},n_{D};\;\theta \right) & =n_{C}^{1+\lambda \theta }\exp\left(\theta v_{C}\left(n_{C},n_{D}\right)\right),\\ G_{D}\left(n_{C},n_{D};\;\theta \right) & =n_{D}^{1+\lambda \theta }\exp\left(\theta v_{D}\left(n_{C},n_{D}\right)\right). \end{array} \end{array}$$

Under weak selection, the first-order term of each rate in Eq. **12** is simply the sum of the corresponding first-order terms from the pure game-dynamic case (Eq. **5**) and the pure complex-contagion case (Eq. **10**).

We can understand how game dynamics and complex contagion collectively drive evolution on hypergraphs by analyzing the probability that a focal defector switches to cooperation within a hyperedge $\left(D,n_{C},n_{D}\right)$, namely $F_{C}/\left(F_{C}+F_{D}\right)$. A Taylor expansion of this expression in the selection strength θ produces the following intuitive expression:

[13] $$\begin{array}{c} \begin{array}{cc} & \frac{F_{C}}{F_{C}+F_{D}}=\frac{n_{C}^{1+\lambda \theta }e^{\theta u_{C}}}{n_{C}^{1+\lambda \theta }e^{\theta u_{C}}+n_{D}^{1+\lambda \theta }e^{\theta u_{D}}}\\ & \approx \frac{n_{C}}{\ell -1}+\theta \;\frac{n_{C}n_{D}}{{\left(\ell -1\right)}^{2}}\left[\left(u_{C}-u_{D}\right)+\lambda \ln\left(\frac{n_{C}}{n_{D}}\right)\right]. \end{array} \end{array}$$

Here the zeroth-order term $n_{C}/\left(\ell -1\right)$ recovers the classical voter model—namely, without selection the focal defector adopts cooperation with a probability equal to the fraction of cooperators among the other individuals in the hyperedge. The strength of selection θ enters into the first-order term, whose components each have clear interpretations. The prefactor $n_{C}n_{D}/{\left(\ell -1\right)}^{2}$ represents the diversity of types in the hyperedge: It is the variance of a Bernoulli random variable with parameter $n_{C}/\left(\ell -1\right)$. This prefactor reflects the fundamental result of Price’s equation (46): the rate of change of a type’s frequency scales with its instantaneous diversity. The term $u_{C}-u_{D}$ represents the payoff difference between a cooperator and a defector, which is the source of payoff-driven change; and the term $\lambda \ln(n_{C}/n_{D})$ represents the effect of the payoff-neutral complex contagion, which depends on the skew in strategy frequencies [quantified by $\ln(n_{C}/n_{D})$] modulated by form of complexity, λ. There is a corresponding, intuitive decomposition of the fixation probability, $\rho_{C}$, into mechanistically distinct additive components (Eq. **38** in *Materials and Methods*).

We can now ask how complex contagion modifies the threshold for cooperation when higher-order interactions shape both payoff and inheritance. Focusing on ℓ=3, we compute the critical benefit-to-cost ratio ${\left(b/c\right)}^{*}$ for both the donation game and the public goods game as a function of the complexity parameter λ. Fig. 4*C* shows that ${\left(b/c\right)}^{*}$ increases monotonically with λ (Eqs. **39** and **40** in *Materials and Methods*). Thus conformity-seeking contagion (λ>0) makes cooperation harder to evolve, whereas novelty-seeking contagion (λ<0) lowers the threshold and promotes cooperation. This monotonicity follows directly from the fact that increasing λ lowers the fixation probability $\rho_{C}$ of a rare cooperator.

We also consider a variant in which the payoffs in Eq. **12** are averaged over the k interactions rather than accumulated (*SI Appendix*, Fig. S3). The qualitative effect of complex contagion is unchanged: Increasing λ still raises the critical ratio ${\left(b/c\right)}^{*}$. Quantitatively, however, the dependence on λ is stronger under averaged payoffs, so the shifts in the cooperation threshold are more pronounced than in the accumulated-payoff case.

## Discussion

Pair approximation, and its generalization to n-site approximations (47), has long served as the workhorse for analyzing dynamical processes on graphs. But many systems exhibit genuine higher-order interactions that cannot be decomposed into pairs, such as ring-exchange interactions in atomic systems (48), social contagion on simplicial structures (49), and the group-level processes analyzed here. The ℓ-hyperedge approximation we develop closes this gap, extending the analytical reach of pair approximation to stochastic processes on regular hypergraphs of arbitrary order.

We have used the approximation to analyze two classes of dynamics on hypergraphs—evolutionary game dynamics and neutral complex contagion—as well as a coupled model that combines them. The main results include the generalization of the classical b/c>k cooperation rule to multiplayer donation games on hypergraphs of arbitrary order; a closed-form threshold benefit-to-cost ratio for the nonlinear public goods game, which remains bounded as k→∞; a closed-form expression for the fixation probability under neutral complex contagion that exposes the role of a single complexity parameter; and a characterization of how conformity and novelty jointly reshape the conditions for the evolution of cooperation.

Before the hyperedge approximation, multiplayer games were studied by assuming each individual joins k+1 games initiated by itself and its k neighbors, with dynamics analyzed by classical pair approximation (50). This construction is equivalent to the case ℓ=2 in our framework (Eqs. **1** and **2**). This approach is limited, though, because it assumes adjacent nodes share no common neighbors, so that groups are built from pairwise links without clustering. This assumption is generally unrealistic, because real-world multiparty interactions typically occur in clustered groups, naturally represented as hyperedges. Because the hyperedge statistics cannot in general be recovered from pairwise-link frequencies alone, the hyperedge approximation is a substantial generalization of pair approximation.

Evolutionary set theory (51) provides an alternative framework for group interactions in structured populations. The two approaches differ in how they treat the group: Evolutionary set theory tracks individuals migrating between groups according to individual fitness, whereas the ℓ-hyperedge approximation treats the groups as static interaction units, with dynamics on the strategies rather than on the group memberships.

Coalescent theory has likewise been applied to evolutionary dynamics on finite structured populations (45, 52–54), and recent advances have extended it to nonlinear cooperation on hypergraphs (36). Coalescent methods are exact, but they require solving a linear system of size $O(N^{\ell +1})$ for each hypergraph (36), so the computational cost grows dramatically with ℓ and becomes infeasible for ℓ≥4. By contrast, the ℓ-hyperedge approximation produces closed-form expressions for fixation probabilities and related quantities.

The limitations of coalescent theory are not only computational. Like most existing analyses of evolutionary dynamics on networks, it rests on a parent-to-offspring inheritance map for each update—a restriction that excludes group-level inheritance entirely. Replacement rules are usually assumed to be distributions over pairs (R,α), where R⊆{1,…,N} is the set of sites updated in a step and α:R→{1,…,N} is an offspring-to-parent map for each site (34). The type at site i∈R becomes $x_{\alpha (i)}$ in the next step. This formulation captures imitation and horizontal transmission, but it assumes that each individual inherits its type from a single “parent.” A natural generalization replaces (R,α) with (R,β), where $\beta :S^{N}\to S^{R}$ assigns a type to each updated individual as a function of the current configuration of the whole population. Replacement rules are the special case of these more general adoption rules in which β is the pullback of an offspring-to-parent map, and the containment is strict. Consider N=3 with a single 3-hyperedge, three types A, B, C, and an update rule under which C is adopted only on joint exposure to A and B. The transition (A,A,B)→(C,A,B) at site 1 cannot arise from any replacement rule, because neither candidate parent has type C. Complex contagion thus lies outside any analysis built on parent-to-offspring inheritance (6, 33, 34, 36).

The examples we have studied generalize classical questions in evolutionary games, but they represent only a small sample from the broad range of applications. Any stochastic process whose outcomes depend on the composition of a group, rather than on pairwise contacts alone, is a candidate for the ℓ-hyperedge approximation. This includes group-mediated transmission processes, as well as evolutionary or cultural dynamics with more than two competing types. In multitype settings, the approximation can be combined with the standard embedded-Markov-chain approach, using pairwise fixation probabilities to derive the stationary distribution across strategies or opinions.

Our results show that novelty-seeking behavior can promote the spread of a rare strategy or opinion, reducing the synergy required for cooperation to emerge (*SI Appendix*, Fig. S4). In real populations, however, novel strategies typically come with additional costs in development and production (55–57). A natural extension is to incorporate these costs into the payoff structure, yielding a tradeoff between the benefits of novelty and its costs, and an optimal bias toward novelty that can itself be characterized within the ℓ-hyperedge framework.

More generally, in group-mediated processes, the same hyperedge that determines a player’s payoff also shapes how that player updates their type, and the two effects compound. The ℓ-hyperedge approximation makes this compounding tractable, and recovers classical results—the b/c>k rule, the voter-model neutral limit, the beta-binomial sampling structure of population genetics—as special cases of a single closure. Whether the same closure extends to inheritance structures with no graphical analog, such as coalition formation or role-based interactions in complex organizations, is an open question.

## Materials and Methods

### ℓ-Hyperedge Approximation and Fixation Probability.

The dynamical system can be represented by the variables $p_{C}$, $q_{\left(n_{C},n_{D}\right)|C}$, and $q_{\left(n_{C},n_{D}\right)|D}$ (for all $n_{C}+n_{D}=\ell -1$). Since the stochastic process of $p_{C}$ is a martingale under neutral drift (θ=0), the dynamics associated with hyperedges, $q_{\left(n_{C},n_{D}\right)|C}$ and $q_{\left(n_{C},n_{D}\right)|D}$, unfold on a much faster timescale than that of the individual strategy $p_{C}$ under weak selection (θ≪1). As a result, $q_{\left(n_{C},n_{D}\right)|C}$ and $q_{\left(n_{C},n_{D}\right)|D}$ rapidly reach equilibrium and can be expressed as polynomial functions of $p_{C}$ and the average degree, k,

[14a] $$q_{\left(n_{C},n_{D}\right)|C}={\left(-1\right)}^{n_{D}}\left(\begin{array}{c} l-1\\ n_{D} \end{array}\right)\frac{\begin{array}{c} \prod_{i=1}^{n_{C}}\left(i+hp_{C}\right)\prod_{j=1}^{n_{D}}\left(hp_{C}-\left(h+j-1\right)\right) \end{array}}{\prod_{i=1}^{l-1}\left(i+h\right)},$$

[14b] $$q_{\left(n_{C},n_{D}\right)|D}={\left(-1\right)}^{n_{D}}\left(\begin{array}{c} l-1\\ n_{D} \end{array}\right)\frac{\begin{array}{c} \prod_{i=1}^{n_{C}}\left(i-1+hp_{C}\right)\prod_{j=1}^{n_{D}}\left(hp_{C}-\left(h+j\right)\right) \end{array}}{\prod_{i=1}^{l-1}\left(i+h\right)},$$

where $h=\left(\ell -1\right)k-\ell$.

A useful way to interpret Eq. **14** is through an analogy with classical population genetics. The equilibrium expressions for $q_{(n_{C},n_{D})|C}$ and $q_{(n_{C},n_{D})|D}$ are not just a convenient closure of the hyperedge dynamics; they belong to the same mathematical family as the stationary mutation–drift distributions that arise in Wright–Fisher theory. In that setting, one thinks of a local allele frequency as fluctuating under genetic drift while being pulled toward an external mean by mutation or, in our case, immigration from a global pool. Here, the analogous quantity is the local frequency of cooperators in a focal hyperedge. The key point is that Eq. **14** can be read as saying that this local frequency is distributed according to a beta distribution whose mean is set by the global frequency of cooperators $p_{C}$, and whose concentration is set by the single parameter h=(ℓ−1)k−ℓ. Thus $p_{C}$ determines the center of the local distribution of cooperators, while h determines how tightly local composition is concentrated around that center.

To see this connection more explicitly, it is helpful to rewrite Eq. **14** in rising-factorial form. Since

[15] $$\begin{array}{c} {\left(-1\right)}^{n_{D}}\prod_{j=1}^{n_{D}}(hp_{C}-\left(h+j-1\right))=\prod_{j=1}^{n_{D}}(h\left(1-p_{C}\right)+j-1), \end{array}$$

we obtain

[16] $$\begin{array}{c} q_{(n_{C},n_{D})|C}=\left(\begin{array}{c} \ell -1\\ n_{D} \end{array}\right)\frac{{\left(1+hp_{C}\right)}_{n_{C}}\;{\left(h\left(1-p_{C}\right)\right)}_{n_{D}}}{{\left(1+h\right)}_{\ell -1}}, \end{array}$$

and similarly

[17] $$\begin{array}{c} q_{(n_{C},n_{D})|D}=\left(\begin{array}{c} \ell -1\\ n_{D} \end{array}\right)\frac{{\left(hp_{C}\right)}_{n_{C}}\;{\left(1+h\left(1-p_{C}\right)\right)}_{n_{D}}}{{\left(1+h\right)}_{\ell -1}}, \end{array}$$

where ${\left(x\right)}_{m}=x\left(x+1\right)\cdots \left(x+m-1\right)$ denotes the rising factorial. These are exactly beta-binomial probabilities that often arise in population genetics. Equivalently, if X denotes a local frequency of cooperators, then

[18] $$\begin{array}{c} X|C∼\mathrm{Beta}\left(1+hp_{C},\;h\left(1-p_{C}\right)\right),\;X|D∼\mathrm{Beta}\left(hp_{C},\;1+h\left(1-p_{C}\right)\right), \end{array}$$

and the number $n_{C}$ of cooperators among the remaining ℓ−1 positions in the hyperedge is obtained by binomial sampling from X. Ignoring for the moment the conditioning on the focal type, the underlying unconditioned form is therefore

[19] $$\begin{array}{c} X∼\mathrm{Beta}(hp_{C},\;h\left(1-p_{C}\right)), \end{array}$$

which is precisely the standard mean–concentration parameterization of a beta distribution from population genetics. In this form, the mean is $E\left[X\right]=p_{C},$ and the variance is $\mathrm{Var}\left(X\right)=p_{C}\left(1-p_{C}\right)/\left(h+1\right).$ Thus $p_{C}$ plays the role of the global or mainland allele frequency, while h plays the role of the compound immigration–drift parameter that controls the magnitude of local fluctuations: Larger h means weaker genetic drift (relative to immigration) and tighter concentration around the mean local frequency.

The conditional law given a focal cooperator has the same interpretation, but with one additional pseudocount on the C side:

[20] $$\begin{array}{c} X|C∼\mathrm{Beta}(1+hp_{C},\;h\left(1-p_{C}\right)). \end{array}$$

Thus conditioning on the focal type does not change the basic interpretation of the distribution: $p_{C}$ still controls the central tendency of local composition, while h still controls the strength of concentration around that center. The extra 1 in the first beta parameter simply reflects the fact that, once the focal individual is known to be a cooperator, the local frequency is biased upward by one effective count on the C side.

In our model, the concentration parameter is h=(ℓ−1)k−ℓ, so both k and ℓ enter only through this single compound quantity. The global frequency $p_{C}$ sets the mean local composition in the hyperedge. The parameters k and ℓ together determine the concentration h around the mean. Increasing the number of hyperedges k increases h, so it strengthens mixing across hyperedges and makes local composition less variable and more tightly tied to the global frequency $p_{C}$; this makes intuitive sense because a focal node is involved in more interactions with other players. Increasing the order ℓ also increases h whenever k≥2, so larger interaction groups likewise increase concentration and reduce drift-like fluctuations in local composition. In this sense, Eq. **14** shows that local hyperedge composition is mathematically identical to a Wright–Fisher mutation–drift equilibrium, reparameterized in terms of the global frequency $p_{C}$ and the effective concentration h=(ℓ−1)k−ℓ. This population-genetic interpretation helps explain why the ℓ-hyperedge approximation takes such a natural and tractable form.

Furthermore, Eq. **14** can be rewritten as $q_{\left(n_{C},n_{D}\right)|C}=\sum_{m=0}^{\ell -1}\alpha_{n_{C},n_{D}}^{\left(m\right)}p_{C}^{m}$ and $q_{\left(n_{C},n_{D}\right)|D}=\sum_{m=0}^{\ell -1}\beta_{n_{C},n_{D}}^{\left(m\right)}p_{C}^{m}$. For $\alpha_{n_{C},n_{D}}^{\left(m\right)}$, we have

[21] $$\begin{array}{c} \begin{array}{cc} \alpha_{n_{C},n_{D}}^{\left(m\right)} & ={\left(-1\right)}^{n_{D}}\left(\begin{array}{c} \ell -1\\ n_{D} \end{array}\right)\\ & \times \frac{\left(\prod_{i=1}^{\ell -1}c_{i}\right)/\left(\begin{array}{c} \sum_{i_{1}=1}^{\ell -1}\sum_{i_{2}=i_{1}+1}^{\ell -1}\\ \cdots \sum_{i_{m}=i_{m-1}+1}^{\ell -1}\frac{1}{c_{i_{1}}\cdots c_{i_{m}}} \end{array}\right)}{\prod_{i=1}^{\ell -1}\left(i+h\right)/h^{m}}, \end{array} \end{array}$$

where ${\left\{c_{n}\right\}}_{n=1}^{\ell -1}=\left\{1,2,\ldots ,n_{C},-h,\ldots ,-\left(h+n_{D}-1\right)\right\}$. For $\beta_{n_{C},n_{D}}^{\left(m\right)}$, when $n_{C}\geq 1$, we have

[22] $$\begin{array}{c} \begin{array}{cc} \beta_{n_{C},n_{D}}^{\left(0\right)} & =0,\\ \beta_{n_{C},n_{D}}^{\left(m\right)} & ={\left(-1\right)}^{n_{D}}\left(\begin{array}{c} \ell -1\\ n_{D} \end{array}\right)\\ & \times \frac{\left(\prod_{i=1}^{\ell -2}d_{i}\right)/\left(\begin{array}{c} \sum_{i_{1}=1}^{\ell -2}\sum_{i_{2}=i_{1}+1}^{\ell -2}\\ \cdots \sum_{i_{m-1}=i_{m-2}+1}^{\ell -2}\frac{1}{d_{i_{1}}\cdots d_{i_{m-1}}} \end{array}\right)}{\prod_{i=1}^{\ell -1}\left(i+h\right)/h^{m}}\;\left(m\geq 1\right), \end{array} \end{array}$$

where ${\left\{d_{n}\right\}}_{n=1}^{\ell -2}=\left\{1,2,\ldots ,n_{C}-1,-\left(h+1\right),\ldots ,-\left(h+n_{D}\right)\right\}$. When $n_{C}=0$, we have

[23] $$\begin{array}{c} \begin{array}{c} \beta_{0,\ell -1}^{\left(m\right)}={\left(-1\right)}^{m}\frac{\left(\prod_{i=1}^{\ell -1}d_{i}\right)/\left(\begin{array}{c} \sum_{i_{1}=1}^{\ell -1}\sum_{i_{2}=i_{1}+1}^{\ell -1}\\ \cdots \sum_{i_{m}=i_{m-1}+1}^{\ell -1}\frac{1}{d_{i_{1}}\cdots d_{i_{m}}} \end{array}\right)}{\prod_{i=1}^{\ell -1}\left(i+h\right)/h^{m}}, \end{array} \end{array}$$

where ${\left\{d_{n}\right\}}_{n=1}^{\ell -1}=\left\{-\left(h+1\right),\ldots ,-\left(h+n_{D}\right)\right\}$.

Applying the diffusion approximation and Dynkin’s Formula (6), the fixation probability that the population reaches the all-C state from the initial condition $p_{C}{\left(t\right)|}_{t=0}=p_{0}$ is

[24] $$\begin{array}{c} \begin{array}{cc} \rho (p_{0}) & =p_{0}+\theta \left(p_{0}\sum_{m=0}^{\ell -1}\frac{1}{\left(m+1)(m+2\right)}{\bar{\tau }}^{\left(m\right)}\right.\\ & \left.-\sum_{m=0}^{\ell -1}\frac{1}{\left(m+1)(m+2\right)}{\bar{\tau }}^{\left(m\right)}p_{0}^{m+2}\right)+O\left(\theta^{2}\right). \end{array} \end{array}$$

Here, ${\bar{\tau }}^{\left(m\right)}$ is determined by $\alpha_{n_{C},n_{D}}^{\left(m\right)}$ and $\beta_{n_{C},n_{D}}^{\left(m\right)}$ and represents the mth polynomial coefficient of the ratio between the expected change and the variance of the change in the frequency of C per unit time, $2E\left(\Delta p_{C}\right)/\;\text{Var}\left(\Delta p_{C}\right)$. We let the initial frequency of C be $p_{0}=1/N$ and take the limit as N→∞, obtaining the fixation probability of C, $\rho_{C}$, as shown in Eq. **4**.

### Fixation Probability Under Evolutionary Game Dynamics.

The expected accumulated payoff of a cooperative neighbor in the hyperedge of type $\left(D,n_{C},n_{D}\right)$ is

[25] $$\begin{array}{c} u_{C}\left(n_{C},n_{D}\right)=a_{n_{C}-1}+\left(k-1\right)\sum_{n_{C}^{\left(1\right)}+n_{D}^{\left(1\right)}=\ell -1}a_{n_{C}^{\left(1\right)}}q_{(n_{C}^{\left(1\right)},n_{D}^{\left(1\right)})|C}, \end{array}$$

where $a_{n_{C}}$ is the payoff of a focal cooperator obtained from a ℓth-order interaction with $n_{C}$ cooperative neighbors. The expected accumulated payoff of a defector neighbor in this hyperedge is

[26] $$\begin{array}{c} u_{D}\left(n_{C},n_{D}\right)=b_{n_{C}}+\left(k-1\right)\sum_{n_{C}^{\left(1\right)}+n_{D}^{\left(1\right)}=\ell -1}b_{n_{C}^{\left(1\right)}}q_{(n_{C}^{\left(1\right)},n_{D}^{\left(1\right)})|D}, \end{array}$$

where $b_{n_{C}}$ is the corresponding payoff of a focal defector. Similarly, the expected accumulated payoff of a cooperative neighbor in the hyperedge of type $\left(C,n_{C},n_{D}\right)$ is

[27] $$\begin{array}{c} v_{C}\left(n_{C},n_{D}\right)=a_{n_{C}}+\left(k-1\right)\sum_{n_{C}^{\left(1\right)}+n_{D}^{\left(1\right)}=\ell -1}a_{n_{C}^{\left(1\right)}}q_{(n_{C}^{\left(1\right)},n_{D}^{\left(1\right)})|C}, \end{array}$$

and the expected accumulated payoff of a defector neighbor in this hyperedge is

[28] $$\begin{array}{c} v_{D}\left(n_{C},n_{D}\right)=b_{n_{C}+1}+\left(k-1\right)\sum_{n_{C}^{\left(1\right)}+n_{D}^{\left(1\right)}=\ell -1}b_{n_{C}^{\left(1\right)}}q_{(n_{C}^{\left(1\right)},n_{D}^{\left(1\right)})|D}. \end{array}$$

In this case, the quantity ${\bar{\tau }}^{\left(m\right)}$ in Eq. **25** becomes ${\bar{\tau }}^{\left(m\right)}={\bar{\mu }}^{\left(m\right)}-{\bar{v}}^{\left(m\right)}$, where

[29] $$\begin{array}{c} \begin{array}{cc} \gamma_{n_{C},n_{D}}^{\left(m\right)} & =\sum_{i=0}^{m}\alpha_{n_{C},n_{D}}^{\left(i\right)}\;\left(0\leq m\leq \ell -1\right),\\ \mu^{\left(m\right)} & =\sum_{\begin{array}{c} n_{C}+n_{D}=\ell -1,\\ n_{D}\geq 1 \end{array}}n_{D}\left(a_{n_{C}}-b_{n_{C}+1}\right)\gamma_{n_{C},n_{D}}^{\left(m\right)}\;\left(0\leq m\leq \ell -2\right),\\ \mu^{\left(\ell -1\right)} & =0,\\ v^{\left(m\right)} & =\sum_{n_{C}+n_{D}=\ell -1}b_{n_{C}}\beta_{n_{C},n_{D}}^{\left(m\right)}-a_{n_{C}}\alpha_{n_{C},n_{D}}^{\left(m\right)}\;\left(0\leq m\leq \ell -1\right),\\ {\bar{\mu }}^{\left(m\right)} & =\frac{N(k-1)}{k\left((\ell -1)k-\ell \right)}\mu^{\left(m\right)},\\ {\bar{v}}^{\left(m\right)} & =\frac{N(k+1)(k-1)}{k}v^{\left(m\right)}. \end{array} \end{array}$$

The quantity ${\bar{\mu }}^{\left(m\right)}$ corresponds to the difference in the expected accumulated payoff between all cooperators surrounded by a defector and all defectors surrounded by a cooperator, while ${\bar{v}}^{\left(m\right)}$ corresponds to the difference in the expected payoff between a single cooperator and a single defector. When the population size is large, the fixation probability of cooperation in this case becomes

[30] $$\begin{array}{c} \rho_{C}=\frac{1}{N}+\frac{\theta }{N}\sum_{m=0}^{\ell -1}\frac{1}{\left(m+1)(m+2\right)}\left({\bar{\mu }}^{\left(m\right)}-{\bar{v}}^{\left(m\right)}\right)+O\left(\theta^{2}\right), \end{array}$$

and $\rho_{C}>1/N$ is equivalent to

[31] $$\begin{array}{c} \sum_{m=0}^{\ell -1}\frac{1}{\left(m+1)(m+2\right)}\left({\bar{\mu }}^{\left(m\right)}-{\bar{v}}^{\left(m\right)}\right)>0. \end{array}$$

### ℓ-Player Donation Game.

When $a_{n_{C}}=-c+n_{C}b$ and $b_{n_{C}}=n_{C}b$, we can prove that

[32] $$\begin{array}{c} \frac{2E(\Delta p_{C})}{\text{Var}(\Delta p_{C})}=\theta N\left(b-kc\right), \end{array}$$

which means ${\bar{\tau }}^{\left(0\right)}={\bar{\mu }}^{\left(0\right)}-{\bar{v}}^{\left(0\right)}=N\left(b-kc\right)$ and ${\bar{\tau }}^{\left(m\right)}={\bar{\mu }}^{\left(m\right)}-{\bar{v}}^{\left(m\right)}=0$ for 1≤m≤ℓ−1. Substituting ${\bar{\tau }}^{\left(m\right)}$ into Eq. **31** gives the b/c>k rule on hypergraphs of order ℓ.

### Relative Success of Cooperation.

The relative success of cooperation over defection is quantified by whether the fixation probability of cooperation exceeds that of defection, i.e., $\rho_{C}>\rho_{D}$. The fixation probability of D, $\rho_{D}$, can be obtained using the complementary relation $\rho_{D}=1-\rho \left(1-1/N\right)$. Then $\rho_{C}>\rho_{D}$ is equivalent to

[33] $$\begin{array}{c} \sum_{m=0}^{\ell -1}\frac{1}{m+1}\left({\bar{\mu }}^{\left(m\right)}-{\bar{v}}^{\left(m\right)}\right)>0, \end{array}$$

in the large population limit.

Take ℓ=2 and ℓ=3 as examples. In the case of ℓ=2 (6), the condition for $\rho_{C}>\rho_{D}$ is

[34] $$\begin{array}{c} \left(k+1\right)a_{0}+\left(k-1\right)a_{1}>\left(k+1\right)b_{0}+\left(k-1\right)b_{1}. \end{array}$$

In the case of ℓ=3, the condition becomes

[35] $$\begin{array}{c} \begin{array}{cc} & \left(8k^{3}-10k^{2}+3k+3\right)a_{0}+\left(8k^{3}-4k^{2}-6\right)a_{1}\\ & +\left(8k^{3}+2k^{2}-3k+3\right)a_{2}>\left(8k^{3}+2k^{2}-3k+3\right)b_{0}\\ & +\left(8k^{3}-4k^{2}-6\right)b_{1}+\left(8k^{3}-10k^{2}+3k+3\right)b_{2}. \end{array} \end{array}$$

### Neutral Complex Contagion.

For ℓ=3, the fixation probability of C is

[36] $$\begin{array}{c} \rho_{C}=\frac{1}{N}+\theta \lambda \left(\ln2\right)\frac{2k-3}{2k-1}\left(-\frac{1}{6}+\frac{1}{2N}-\frac{1}{3N^{2}}\right)+O\left(\theta^{2}\right). \end{array}$$

When N is sufficiently large, $\rho_{C}$ becomes

[37] $$\begin{array}{c} \rho_{C}=\frac{1}{N}-\frac{\theta \lambda \left(\ln\;2\right)\left(2k-3\right)}{6(2k-1)}+O\left(\theta^{2}\right). \end{array}$$

We refer to *SI Appendix* for the analytical derivations of $\rho_{C}$ for arbitrary orders ℓ.

### Complex Contagion with Payoff-Biased Selection.

We calculate the fixation probability of cooperation in the case of evolutionary game dynamics combined with complex contagion for ℓ=3:

[38] $$\begin{array}{c} \rho_{C}=\frac{1}{N}+\frac{\theta }{24k(2k-1)}\left(\begin{array}{l} a_{0}\left(12k^{3}-16k^{2}+3k+3\right)+a_{1}\left(8k^{3}-6k-6\right)\\ +a_{2}\left(4k^{3}+4k^{2}+3k+3\right)-b_{0}\left(12k^{3}-9k+3\right)\\ -b_{1}\left(8k^{3}-8k^{2}+6k-6\right)-b_{2}\left(4k^{3}-4k^{2}+3k+3\right) \end{array}\right)\\ -4\lambda k\left(\text{ln}\;2\right)\left(2k-3\right))+O\left(\theta^{2}\right). \end{array}$$

When c=1, the critical benefit-to-cost ratio in the donation game is

[39] $$\begin{array}{c} {\left(\frac{b}{c}\right)}^{*}=k+\frac{\lambda \left(\ln\;2\right)\left(2k-3\right)}{3\left(2k-1\right)}, \end{array}$$

and the critical benefit-to-cost ratio in the public goods game with benefit factor δ is

[40] $$\begin{array}{c} {\left(\frac{b}{c}\right)}^{*}=\frac{36k^{2}\left(2k-1\right)+12\lambda k\left(\ln\;2\right)\left(2k-3\right)}{\left(k+1\right)\left(\delta^{2}\left(4k^{2}+3\right)+\delta \left(8k^{2}-6\right)+3{\left(2k-1\right)}^{2}\right)}. \end{array}$$

## Supplementary Material

Appendix 01 (PDF)

## Data Availability

There are no data underlying this work.
