# Supplementary material for: Hyperedge approximation for stochastic processes on higher-order networks
Source: Proc Natl Acad Sci U S A. 2026 Aug 5;123(32):e2619078123. doi: 10.1073/pnas.2619078123 (PMC13461965; doi:10.1073/pnas.2619078123)
Supplement: Supplementary file 1 [file pnas.2619078123.sapp.pdf]

Hyperedge approximation for stochastic processes on higher-order networks

Supporting Information

1 ℓ-hyperedge approximation

We consider a population of size N. The population structure is represented by a regular graph of order exactly ℓ and degree k, which means that every individual has exactly k (hyper)edges, each of order ℓ. Note that the (hyper)graph has a fixed order, so that it does not simultaneously contain, eg, edges and 2-simplices. Every individual chooses either strategy C or D and interacts with their neighbors.

We use the ℓ-hyperedge approximation, a generalization of the classical pair approximation (1), to analyze stochastic processes on hypergraphs of any given order ℓ. Let p_C (resp. p_D) denote the frequency of C-individuals (resp. D-individuals) in the population. Let p_{C,n_C,n_D} (resp. p_{D,n_C,n_D}) denote the frequency of the hyperedges where the focal individual is a C-individual (resp. a D-individual) and has n_C C-neighbors and n_D D-neighbors. We consistently have $n_C + n_D = \ell - 1$. Let $q_{(n_C,n_D)|C}$ (resp. $q_{(n_C,n_D)|D}$) denote the conditional probability that, given the focal individual chooses strategy C (resp. strategy D), there are n_C C-neighbors and n_D D-neighbors in the hyperedge. We have the following identities:

$$p_C + p_D = 1, \tag{1a}$$

$$p_{C,n_C,n_D} = p_C \cdot q_{(n_C,n_D)|C}, \; p_{D,n_C,n_D} = p_D \cdot q_{(n_C,n_D)|D}, \tag{1b}$$

$$\sum_{n_C+n_D=\ell-1} q_{(n_C,n_D)|C} = 1, \quad \sum_{n_C+n_D=\ell-1} q_{(n_C,n_D)|D} = 1, \tag{1c}$$

$$n_D p_{C,n_C,n_D} = (n_C+1) p_{D,n_C+1,n_D-1} \quad (n_D \geq 1)\,. \tag{1d}$$

Equation 1d follows from the fact that a hyperedge containing $n_C + 1$ C-individuals and n_D D-individuals indicates $n_C + 1$ hyperedges of type (C, n_C, n_D) and n_D hyperedges of type $(D, n_C + 1, n_D - 1)$. By Eq. 1d, we have

$$p_C n_D q_{(n_C,n_D)|C} = p_D \cdot (n_C+1)\, q_{(n_C+1,n_D-1)|D}. \tag{2}$$

Summing both sides of Eq. 2 over n_D from 1 to $\ell - 1$ gives

$$p_C \sum_{n_C+n_D=\ell-1} n_D q_{(n_C,n_D)|C} = p_D \sum_{n_C+n_D=\ell-1} n_C q_{(n_C,n_D)|D}. \tag{3}$$

Note that we introduce $4\ell + 2$ variables and obtain $3\ell + 2$ identities, so the system can be described by ℓ independent variables, p_C and $q_{(n_C,n_D)|C}$ ($n_C = 1, ..., \ell - 1$).

1.1 Updating a D-individual

At each time step, a randomly selected individual has a chance to update their strategy. We first analyze the case where a randomly selected D-individual updates their strategy to C. Let k_{n_C,n_D} denote the number of hyperedges where there exist n_C C-neighbors and n_D D-neighbors. We have $\sum_{n_C+n_D=\ell-1} k_{n_C,n_D} = k$. Let $\mathbf{k} = (k_{0,\ell-1}, ..., k_{\ell-1,0})$ denote the neighborhood configuration. The probability that the configuration $\mathbf{k}$ occurs follows a multinomial distribution,

$$\mathcal{M}_D(\mathbf{k}) = \frac{k!}{\prod_{n_C+n_D=\ell-1} k_{n_C,n_D}!} \prod_{n_C+n_D=\ell-1} q_{(n_C,n_D)|D}^{k_{n_C,n_D}}. \tag{4}$$

Let $F_C(n_C, n_D; \theta)$ (resp. $F_D(n_C, n_D; \theta)$) denote the rate that a focal D-individual adopts strategy C (resp. strategy D) within the hyperedge (D, n_C, n_D) during the updating process. Here, the parameter θ quantifies the intensity of selection. The probability that the focal D-individual switches their strategy to C under configuration $\mathbf{k}$ is obtained

by averaging over all hyperedges:

$$\mathcal{I}_D(\mathbf{k}) = \frac{\sum_{n_C+n_D=\ell-1} k_{n_C,n_D} F_C(n_C,n_D;\theta)}{\sum_{n_C+n_D=\ell-1} k_{n_C,n_D} \left(F_C(n_C,n_D;\theta) + F_D(n_C,n_D;\theta)\right)}, \tag{5}$$

Then, p_C increases by $1/N$ with probability

$$\mathbb{P}\left(\Delta p_C = \frac{1}{N}\right) = p_D \sum_{k_{0,\ell-1}+\ldots+k_{\ell-1,0}=k} \mathcal{M}_D(\mathbf{k})\mathcal{I}_D(\mathbf{k}). \tag{6}$$

Accordingly, $p_{C,(n_C,n_D)}$ increases by $k_{n_C,n_D}/\left(Nk/\ell\right)$ with probability

$$\mathbb{P}\left(\Delta p_{C,(n_C,n_D)} = \frac{\ell k_{n_C,n_D}}{Nk}\right) = p_D \mathcal{M}_D(\mathbf{k})\mathcal{I}_D(\mathbf{k}). \tag{7}$$

1.2 Updating a C-individual

We then analyze the case where a randomly selected C-individual updates their strategy to D. The probability that the configuration $\mathbf{k}$ occurs in this case is

$$\mathcal{M}_C(\mathbf{k}) = \frac{k!}{\prod_{n_C+n_D=\ell-1} k_{n_C,n_D}!} \prod_{n_C+n_D=\ell-1} q_{(n_C,n_D)|C}^{k_{n_C,n_D}}. \tag{8}$$

Let $G_C(n_C,n_D;\theta)$ (resp. $G_D(n_C,n_D;\theta)$) denote the rate that a focal C-individual adopts strategy C (resp. strategy D) within the hyperedge (C,n_C,n_D) during the updating process. The probability that the focal C-individual switches their strategy to D under configuration $\mathbf{k}$ is

$$\mathcal{I}_C(\mathbf{k}) = \frac{\sum_{n_C+n_D=\ell-1} k_{n_C,n_D} G_D(n_C,n_D;\theta)}{\sum_{n_C+n_D=\ell-1} k_{n_C,n_D} \left(G_C(n_C,n_D;\theta) + G_D(n_C,n_D;\theta)\right)}. \tag{9}$$

Then, p_C decreases by $1/N$ with probability

$$\mathbb{P}\left(\Delta p_C = -\frac{1}{N}\right) = p_C \sum_{k_{0,\ell-1}+\ldots+k_{\ell-1,0}=k} \mathcal{M}_C(\mathbf{k})\mathcal{I}_C(\mathbf{k}). \tag{10}$$

Accordingly, $p_{C,(n_C,n_D)}$ decreases by $k_{n_C,n_D}/\left(Nk/\ell\right)$ with probability

$$\mathbb{P}\left(\Delta p_{C,(n_C,n_D)} = -\frac{\ell k_{n_C,n_D}}{Nk}\right) = p_C \mathcal{M}_C(\mathbf{k})\mathcal{I}_C(\mathbf{k}). \tag{11}$$

We assume that the evolutionary dynamics of strategies follow a martingale without selection ($\theta = 0$), indicating that the expected change in frequency is unbiased for both strategies C and D at every time step without selection (given the previous strategy frequency p_C). Then, we have the identity

$$\frac{F_C(n_C,n_D;0)}{F_D(n_C,n_D;0)} = \frac{G_C(n_C,n_D;0)}{G_D(n_C,n_D;0)} = \frac{n_C}{n_D}. \tag{12}$$

1.3 Differential equations of strategies on nodes and along hyperedges

As only one replacement event occurs in one unit of time, we have the time derivative of p_C

$$\dot{p}_C = \frac{1}{N} \cdot \mathbb{P}\left(\Delta p_C = \frac{1}{N}\right) + \frac{1}{N} \cdot \mathbb{P}\left(\Delta p_C = -\frac{1}{N}\right). \tag{13}$$

By Eqs. 3 and 12, we obtain that $\dot{p}_C = 0$ without selection (i.e. $\theta = 0$). Therefore, under weak selection (i.e. $\theta \ll 1$), we have

$$\dot{p}_C = \frac{\theta}{N}\left\{\frac{\begin{pmatrix} p_D \sum_{n_C+n_D=\ell-1} q_{(n_C,n_D)|D}\partial_\theta F_C(n_C,n_D;0) \\ -p_C \sum_{n_C+n_D=\ell-1} q_{(n_C,n_D)|C}\partial_\theta G_D(n_C,n_D;0)\end{pmatrix}}{\ell-1} - \frac{1}{k(\ell-1)^2}\left[\begin{pmatrix} p_D \sum_{n_C+n_D=\ell-1} q_{(n_C,n_D)|D} n_C (\partial_\theta F_C(n_C,n_D;0)+\partial_\theta F_D(n_C,n_D;0)) \\ -p_C \sum_{n_C+n_D=\ell-1} q_{(n_C,n_D)|C} n_D (\partial_\theta G_C(n_C,n_D;0)+\partial_\theta G_D(n_C,n_D;0))\end{pmatrix}\right.\right.$$
$$\left.\left. + \begin{pmatrix} (k-1)p_D \sum_{n_C^{(1)}+n_D^{(1)}=\ell-1} n_C^{(1)} q_{n_C^{(1)},n_D^{(1)}|D} \\ \times \sum_{n_C^{(2)}+n_D^{(2)}=\ell-1} q_{n_C^{(2)},n_D^{(2)}|D}\left(\partial_\theta F_C\left(n_C^{(2)},n_D^{(2)};0\right)+\partial_\theta F_D\left(n_C^{(2)},n_D^{(2)};0\right)\right) \\ -(k-1)p_C \sum_{n_C^{(1)}+n_D^{(1)}=\ell-1} n_D^{(1)} q_{n_C^{(1)},n_D^{(1)}|C} \\ \times \sum_{n_C^{(2)}+n_D^{(2)}=\ell-1} q_{n_C^{(2)},n_D^{(2)}|C}\left(\partial_\theta G_C\left(n_C^{(2)},n_D^{(2)};0\right)+\partial_\theta G_D\left(n_C^{(2)},n_D^{(2)};0\right)\right)\end{pmatrix}\right]\right\} + O\left(\theta^2\right). \tag{14}$$

To simplify this equation, we first prove the following two identities:

$$\begin{aligned} p_D \sum_{n_C+n_D=\ell-1} q_{(n_C,n_D)|D}\partial_\theta F_C(n_C,n_D;0) &= p_C \sum_{n_C+n_D=\ell-1} \frac{n_D}{n_C+1} q_{(n_C,n_D)|C}\partial_\theta F_C(n_C+1,n_D-1;0), \\ p_D \sum_{n_C+n_D=\ell-1} q_{(n_C,n_D)|D} n_C \partial_\theta F_C(n_C,n_D;0) &= p_C \sum_{n_C+n_D=\ell-1} q_{(n_C,n_D)|C} n_D \partial_\theta F_C(n_C+1,n_D-1;0). \end{aligned} \tag{15}$$

For the first identity, by Eq. 2, we have

$$\begin{aligned} p_D \sum_{n_C+n_D=\ell-1} q_{(n_C,n_D)|D}\partial_\theta F_C(n_C,n_D;0) &= p_D \sum_{n_C=1}^{\ell-1} q_{(n_C,n_D)|D} n_C \frac{\partial_\theta F_C(n_C,n_D;0)}{n_C} \\ &= p_D \sum_{n_C=0}^{\ell-2} q_{(n_C+1,n_D-1)|D}(n_C+1)\frac{\partial_\theta F_C(n_C+1,n_D-1;0)}{n_C+1} \\ &= p_C \sum_{n_C=0}^{\ell-2} q_{(n_C,n_D)|C} n_D \frac{\partial_\theta F_C(n_C+1,n_D-1;0)}{n_C+1} \\ &= p_C \sum_{n_C+n_D=\ell-1} n_D q_{(n_C,n_D)|C}\frac{\partial_\theta F_C(n_C+1,n_D-1;0)}{n_C+1} \end{aligned}. \tag{16}$$

We can use a similar idea to prove the second identity.

Define

$$z_C := \sum_{n_C+n_D=\ell-1} n_D q_{(n_C,n_D)|C},\ z_D = \sum_{n_C+n_D=\ell-1} n_C q_{(n_C,n_D)|D}. \tag{17}$$

Using Eqs. 15 and 17, the time derivative of p_C (Eq. 18) can be simplified as

$$\begin{aligned}\dot{p}_C = \frac{\theta}{N}\Bigg\{ & \frac{p_C \sum_{n_C+n_D=\ell-1} q_{(n_C,n_D)|C}\left(\partial_\theta F_C\left(n_C+1,n_D-1;0\right)\frac{n_D}{n_C+1}-\partial_\theta G_D\left(n_C,n_D;0\right)\right)}{\ell-1} \\ & -\frac{1}{k(\ell-1)^2}\left[\left(p_C\sum_{n_C+n_D=\ell-1} q_{(n_C,n_D)|C}n_D\left(\begin{matrix}\partial_\theta F_C(n_C+1,n_D-1;0)+\partial_\theta F_D(n_C+1,n_D-1;0)\\ -\partial_\theta G_C(n_C,n_D;0)-\partial_\theta G_D(n_C,n_D;0)\end{matrix}\right)\right)\right. \\ & \left.+\left(\begin{matrix}(k-1)p_D z_D\sum_{n_C+n_D=\ell-1} q_{(n_C,n_D)|D}(\partial_\theta F_C(n_C,n_D;0)+\partial_\theta F_D(n_C,n_D;0))\\ -(k-1)p_C z_C\sum_{n_C+n_D=\ell-1} q_{(n_C,n_D)|C}(\partial_\theta G_C(n_C,n_D;0)+\partial_\theta G_D(n_C,n_D;0))\end{matrix}\right)\right]\Bigg\}+O\left(\theta^2\right)\end{aligned} \quad . \tag{18}$$

The time derivative of $p_{C,(n_C,n_D)}$ (for $n_C=0,...,\ell-1$) is

$$\begin{aligned}\dot{p}_{C,(n_C,n_D)} &= p_D\sum_{\mathbf{k}}\frac{\ell k_{n_C,n_D}}{Nk}\mathcal{M}_D(\mathbf{k})\frac{\sum_{n_C^{(1)}+n_D^{(1)}=\ell-1} k_{n_C^{(1)},n_D^{(1)}}n_C^{(1)}}{k(\ell-1)} \\ &\quad - p_C\sum_{\mathbf{k}}\frac{\ell k_{n_C,n_D}}{Nk}\mathcal{M}_C(\mathbf{k})\frac{\sum_{n_C^{(1)}+n_D^{(1)}=\ell-1} k_{n_C^{(1)},n_D^{(1)}}n_D^{(1)}}{k(\ell-1)}+O\left(\theta\right) \\ &= \frac{\ell p_D}{Nk(\ell-1)}q_{(n_C,n_D)|D}\left(n_C+(k-1)\sum_{n_C^{(1)}+n_D^{(1)}=\ell-1} n_C^{(1)}q_{n_C^{(1)},n_D^{(1)}|D}\right) \\ &\quad - \frac{\ell p_C}{Nk(\ell-1)}q_{(n_C,n_D)|C}\left(n_D+(k-1)\sum_{n_C^{(1)}+n_D^{(1)}=\ell-1} n_D^{(1)}q_{n_C^{(1)},n_D^{(1)}|C}\right)+O\left(\theta\right)\end{aligned} \quad . \tag{19}$$

Under weak selection, the frequencies of hyperedges, p_{C,n_C,n_D}, reach the equilibrium much faster than the global strategy frequency, p_C, that is, p_{C,n_C,n_D} can be expressed as a function of p_C. Define

$$x_i := q_{\ell-i,i-1|C} - q_{\ell-i,i-1|D}. \tag{20}$$

By Eq. 1, we have $\sum_{i=1}^{\ell} x_i = 0$ and $p_C z_C = p_D z_D$. Using Eqs. 2 and 3, the equilibrium can be obtained by solving the following system of equations:

$$\left\{\begin{aligned} p_C\cdot 1\cdot q_{\ell-2,1|C} - p_C\cdot 0\cdot q_{\ell-1,0|C} &= p_C(k-1)x_1 z_C, \\ p_C\cdot 2\cdot q_{\ell-3,2|D} - p_C\cdot 1\cdot q_{\ell-2,1|C} &= p_C(k-1)x_2 z_C, \\ &\vdots \\ p_C\cdot(\ell-1)\cdot q_{0,\ell-1|C} - p_C\cdot(\ell-2)\cdot q_{1,\ell-2|C} &= p_C(k-1)x_{\ell-1}z_C, \\ x_1\cdot(\ell-1)\cdot(1-p_C) &= (\ell-1)\cdot(1-p_C)\cdot q_{\ell-1,0|C} - 1\cdot p_C\cdot q_{\ell-2,1|C}, \\ x_2\cdot(\ell-2)\cdot(1-p_C) &= (\ell-2)\cdot(1-p_C)\cdot q_{\ell-2,1|C} - 2\cdot p_C\cdot q_{\ell-3,2|C}, \\ &\vdots \\ x_{\ell-1}\cdot 1\cdot(1-p_C) &= 1\cdot(1-p_C)\cdot q_{1,\ell-2|C} - (\ell-1)\cdot p_C\cdot q_{0,\ell-1|C}, \\ p_C n_D q_{(n_C,n_D)|C} &= p_D\left(n_C+1\right)q_{(n_C,n_D)|D} \quad (n_D=1,...,\ell-1), \\ \sum_{n_C+n_D=\ell-1} q_{(n_C,n_D)|C} &= 1. \end{aligned}\right. \tag{21}$$

As a result, $q_{(n_C,n_D)|C}$ and $q_{(n_C,n_D)|D}$ for all n_C, n_D with $n_C+n_D=\ell-1$ can be expressed as functions of p_C and k.

Let $h=(\ell-1)k-\ell$. We derive the closed-form solution for the linear system in Eq. 21

$$q_{(n_C,n_D)|C}=(-1)^{n_D}\binom{\ell-1}{n_D}\frac{\prod_{i=1}^{n_C}(i+hp_C)\prod_{j=1}^{n_D}(hp_C-(h+j-1))}{\prod_{i=1}^{\ell-1}(i+h)}, \tag{22a}$$

$$q_{(n_C,n_D)|D}=(-1)^{n_D}\binom{\ell-1}{n_D}\frac{\prod_{i=1}^{n_C}(i-1+hp_C)\prod_{j=1}^{n_D}(hp_C-(h+j))}{\prod_{i=1}^{\ell-1}(i+h)}. \tag{22b}$$

We note that $q_{(n_C,n_D)|C}$ and $q_{(n_C,n_D)|D}$ are polynomial functions of p_C with degree at most $\ell-1$ for all n_C,n_D. It follows that $q_{(n_C,n_D)|C}$ and $q_{(n_C,n_D)|D}$ take the following polynomial forms:

$$q_{(n_C,n_D)|C}=\sum_{m=0}^{\ell-1}\alpha_{n_C,n_D}^{(m)}p_C^m,\ q_{(n_C,n_D)|D}=\sum_{m=0}^{\ell-1}\beta_{n_C,n_D}^{(m)}p_C^m. \tag{23}$$

Let $\{c_n\}_{n=1}^{\ell-1}=\{1,2,...,n_C,-h,...,-(h+n_D-1)\}$. Comparing Eq. 23 with Eq. 22a gives

$$\alpha_{n_C,n_D}^{(m)}=(-1)^{n_D}\binom{\ell-1}{n_D}\frac{h^m\left(\prod_{i=1}^{\ell-1}c_i\right)/\left(\sum_{i_1=1}^{\ell-1}\sum_{i_2=i_1+1}^{\ell-1}\cdots\sum_{i_m=i_{m-1}+1}^{\ell-1}\frac{1}{c_{i_1}\dots c_{i_m}}\right)}{\prod_{i=1}^{\ell-1}(i+h)}. \tag{24}$$

For $\beta_{n_C,n_D}^{(m)}$, if $n_C\geq 1$ (i.e. $n_D\leq\ell-2$), define a sequence $\{d_n\}_{n=1}^{\ell-2}=\{1,2,...,n_C-1,-(h+1),...,-(h+n_D)\}$. In this case,

$$\begin{aligned}&\beta_{n_C,n_D}^{(0)}=0,\\ &\beta_{n_C,n_D}^{(m)}=(-1)^{n_D}\binom{\ell-1}{n_D}\frac{h^m\left(\prod_{i=1}^{\ell-2}d_i\right)/\left(\sum_{i_1=1}^{\ell-2}\sum_{i_2=i_1+1}^{\ell-2}\cdots\sum_{i_{m-1}=i_{m-2}+1}^{\ell-2}\frac{1}{d_{i_1}\dots d_{i_{m-1}}}\right)}{\prod_{i=1}^{\ell-1}(i+h)}\quad(m\geq 1).\end{aligned} \tag{25}$$

If $n_C=0$ (i.e. $n_D=\ell-1$), define a sequence $\{d_n\}_{n=1}^{\ell-1}=\{-(h+1),...,-(h+n_D)\}$. Then, we have

$$\beta_{0,l-1}^{(m)}=(-1)^m\frac{h^m\left(\prod_{i=1}^{\ell-1}d_i\right)/\left(\sum_{i_1=1}^{\ell-1}\sum_{i_2=i_1+1}^{\ell-1}\cdots\sum_{i_m=i_{m-1}+1}^{\ell-1}\frac{1}{d_{i_1}\dots d_{i_m}}\right)}{\prod_{i=1}^{\ell-1}(i+h)}. \tag{26}$$

Plugging k back in, we obtain the results expressed as functions of p_C and k.

Plugging Eq. 22 into Eq. 18 gives

$$\mathbb{E}(\Delta p_C)=\dot{p}_C=\theta H(p_C;k)+O\left(\theta^2\right), \tag{27}$$

where H is a polynomial function of p_C.

2 Diffusion approximation and fixation probability

Equation 27 gives the expected change in the frequency of strategy C. We turn to calculate the variance of Δp_C. We have

$$\begin{aligned}\mathrm{Var}(\Delta p_C)&=\mathbb{E}\left(\Delta p_C^2\right)-(\mathbb{E}(\Delta p_C))^2\\ &=\frac{1}{N^2}\left(\mathbb{P}\left(\Delta p_C=\frac{1}{N}\right)+\mathbb{P}\left(\Delta p_C=-\frac{1}{N}\right)\right)\Big|_{\theta=0}+O(\theta).\\ &=\frac{2}{N^2(\ell-1)}\frac{p_C(1-p_C)[(\ell-1)k-\ell]}{k-1}\end{aligned} \tag{28}$$

We apply Dynkin's Formula to calculate the fixation probability of strategy C, the probability that the population reaches the all-C absorbing state starting from the initial condition $p_C\,(t=0)=p_0$. Specifically, the fixation probability $\rho(p_0)$ satisfies the following differential equation

$$\mathbb{E}(\Delta p_C)|_{p_C=p_0}\frac{d\rho(p_0)}{dp_0}+\frac{\mathrm{Var}(\Delta p_C)|_{p_C=p_0}}{2}\frac{d^2\rho(p_0)}{dp_0^2}=0. \tag{29}$$

To solve Eq. 29, we introduce the quantity

$$G(p_C)=\exp\left(-\int\frac{2\mathbb{E}(\Delta p_C)}{\mathrm{Var}(\Delta p_C)}\,dp_C\right). \tag{30}$$

We notice that, for any $n_D\geq 1$, the quantity $q_{(n_C,n_D)|C}$ has a factor $1-p_C$. It follows that

$$\frac{q_{(n_C,n_D)|C}}{1-p_C}=\sum_{m=0}^{\ell-2}\gamma^{(m)}_{n_C,n_D}p_C^m, \tag{31}$$

where $\gamma^{(m)}_{n_C,n_D}=\sum_{i=0}^{m}\alpha^{(i)}_{n_C,n_D}$. Then, by Eq. 22, we obtain that the ratio between $\mathbb{E}(\Delta p_C)$ and $\mathrm{Var}(\Delta p_C)$ can also be expressed as a polynomial function of p_C,

$$\frac{2\mathbb{E}(\Delta p_C)}{\mathrm{Var}(\Delta p_C)}=\theta\sum_{m=0}^{\ell-1}\overline{\tau}^{(m)}p_C^m, \tag{32}$$

where

$$\begin{aligned}
\mu^{(m)}&:=\sum_{n_C+n_D=\ell-1}\left(\partial_\theta F_C\left(n_C+1,n_D-1;0\right)\frac{n_D}{n_C+1}-\partial_\theta G_D\left(n_C,n_D;0\right)\right)\gamma^{(m)}_{n_C,n_D}\quad(0\leq m\leq\ell-2),\ \mu^{(\ell-1)}:=0,\\
\xi^{(m)}&:=\sum_{n_C+n_D=\ell-1}\left(\begin{smallmatrix}\partial_\theta F_C(n_C+1,n_D-1;0)+\partial_\theta F_D(n_C+1,n_D-1;0)\\-\partial_\theta G_C(n_C,n_D;0)-\partial_\theta G_D(n_C,n_D;0)\end{smallmatrix}\right)\gamma^{(m)}_{n_C,n_D}\quad(0\leq m\leq\ell-2),\ \xi^{(\ell-1)}:=0,\\
v^{(m)}&:=\sum_{n_C+n_D=\ell-1}\left(\partial_\theta F_C\left(n_C,n_D;0\right)+\partial_\theta F_D\left(n_C,n_D;0\right)\right)\beta^{(m)}_{n_C,n_D}-\left(\partial_\theta G_C\left(n_C,n_D;0\right)+\partial_\theta G_D\left(n_C,n_D;0\right)\right)\alpha^{(m)}_{n_C,n_D},\\
\overline{\mu}^{(m)}&:=\frac{N(k-1)}{(\ell-1)k-\ell}\mu^{(m)},\\
\overline{\xi}^{(m)}&:=\frac{N(k-1)}{k(\ell-1)\left[(\ell-1)k-\ell\right]}\xi^{(m)},\\
\overline{v}^{(m)}&:=\frac{N(k-1)}{k(\ell-1)}v^{(m)},\\
\overline{\tau}^{(m)}&:=\overline{\mu}^{(m)}-\overline{\xi}^{(m)}-\overline{v}^{(m)}.
\end{aligned} \tag{33}$$

Then, the fixation probability of C is given by

$$\rho(p_0)=\frac{\int_0^{p_0}G(p_C)\,dp_C}{\int_0^1 G(p_C)\,dp_C}=p_0+\theta\left(p_0\sum_{m=0}^{\ell-1}\frac{1}{(m+1)(m+2)}\overline{\tau}^{(m)}-\sum_{m=0}^{\ell-1}\frac{1}{(m+1)(m+2)}\overline{\tau}^{(m)}p_0^{m+2}\right)+O\left(\theta^2\right). \tag{34}$$

Define $\rho_C:=\rho\left(1/N\right)$ as the fixation probability of a single C-individual in a population of $N-1$ D-individuals. In the large-population limit (i.e. $N\gg 1$), the absolute success of cooperation, $\rho_C>1/N$, is equivalent to

$$\sum_{m=0}^{\ell-1}\frac{1}{(m+1)(m+2)}\overline{\tau}^{(m)}>0. \tag{35}$$

We can also calculate the fixation probability of a single D-individual, ρ_D, by the relation $\rho_D = 1 - \rho(1 - 1/N)$, and the relative success of cooperation, $\rho_C > \rho_D$, is equivalent to

$$\sum_{m=0}^{\ell-1} \frac{1}{m+1} \overline{\tau}^{(m)} > 0. \tag{36}$$

3 Evolutionary game dynamics

The above analytical framework allows us to analyze a broad range of stochastic processes on hypergraphs. We begin with the evolutionary game dynamics on hypergraphs. In this scenario, every individual either chooses cooperation (C) or defection (D) and interacts with their neighbors via hyperedges. The interaction is described by an ℓ-player game with the most general payoff matrix as shown in Table 1.

Table 1: ℓ-player game on hyperedges

n_C	0	1	...	$\ell-1$
C	a_0	a_1	...	$a_{\ell-1}$
D	b_0	b_1	...	$b_{\ell-1}$

Here, a_{n_C} (resp. b_{n_C}) represents the payoffs of the cooperative (resp. defective) focal player, and n_C represents the number of cooperative neighbors in the hyperedge. For convenience, we additionally define $a_{-1} = b_{-1} = a_\ell = b_\ell = 0$.

We also begin by considering the update of a defector. The payoff of a cooperative neighbor in the hyperedge of type (D, n_C, n_D) is

$$u_C(n_C, n_D) = a_{n_C-1} + (k-1) \sum_{n_C^{(1)}+n_D^{(1)}=\ell-1} a_{n_C^{(1)}} q_{(n_C^{(1)}, n_D^{(1)})|C}, \tag{37}$$

and the payoff of a defective neighbor in this hyperedge is

$$u_D(n_C, n_D) = b_{n_C} + (k-1) \sum_{n_C^{(1)}+n_D^{(1)}=\ell-1} b_{n_C^{(1)}} q_{(n_C^{(1)}, n_D^{(1)})|D}. \tag{38}$$

The fitness of each cooperative and defective neighbor is $f_C(n_C, n_D) = 1 + \theta u_C(n_C, n_D)$ and $f_D(n_C, n_D) = 1 + \theta u_D(n_C, n_D)$ under weak selection.

Likewise, when updating a cooperator, the payoff of a cooperative neighbor in the hyperedge of type (C, n_C, n_D) is

$$v_C(n_C, n_D) = a_{n_C} + (k-1) \sum_{n_C^{(1)}+n_D^{(1)}=\ell-1} a_{n_C^{(1)}} q_{(n_C^{(1)}, n_D^{(1)})|C}, \tag{39}$$

and the payoff of a defective neighbor in this hyperedge is

$$v_D(n_C, n_D) = b_{n_C+1} + (k-1) \sum_{n_C^{(1)}+n_D^{(1)}=\ell-1} b_{n_C^{(1)}} q_{(n_C^{(1)}, n_D^{(1)})|D}. \tag{40}$$

The fitness of each cooperative and defective neighbor is $g_C(n_C, n_D) = 1 + \theta v_C(n_C, n_D)$ and $g_D(n_C, n_D) = 1 + \theta v_D(n_C, n_D)$ under weak selection. In this case,

$$\begin{aligned} &F_C(n_C, n_D; \theta) = n_C f_C(n_C, n_D),\ F_D(n_C, n_D; \theta) = n_D f_D(n_C, n_D), \\ &G_C(n_C, n_D; \theta) = n_C g_C(n_C, n_D),\ G_D(n_C, n_D; \theta) = n_D g_D(n_C, n_D). \end{aligned} \tag{41}$$

and

$$\begin{aligned} &\partial_\theta F_C(n_C, n_D; 0) = n_C u_C(n_C, n_D),\ \partial_\theta G_C(n_C, n_D; 0) = n_C v_C(n_C, n_D), \\ &\partial_\theta F_D(n_C, n_D; 0) = n_D u_D(n_C, n_D),\ \partial_\theta G_D(n_C, n_D; 0) = n_D v_D(n_C, n_D). \end{aligned} \tag{42}$$

Plugging Eq. 42 into Eq. 18 gives

$$\dot{p}_C = \frac{\theta}{N} \left\{ \frac{\begin{pmatrix} p_D \sum_{n_C+n_D=\ell-1} q_{(n_C,n_D)|D} n_C u_C(n_C,n_D) \\ -p_C \sum_{n_C+n_D=\ell-1} q_{(n_C,n_D)|C} n_D v_D(n_C,n_D) \end{pmatrix}}{\ell - 1} - \frac{1}{k(\ell-1)^2} \left[\begin{pmatrix} p_D \sum_{n_C+n_D=\ell-1} q_{(n_C,n_D)|D} n_C (n_C u_C(n_C,n_D) + n_D u_D(n_C,n_D)) \\ -p_C \sum_{n_C+n_D=\ell-1} q_{(n_C,n_D)|C} n_D (n_C v_C(n_C,n_D) + n_D v_D(n_C,n_D)) \end{pmatrix} \right.\right. .$$
$$\left.\left. + \begin{pmatrix} (k-1) p_D z_D \sum_{n_C+n_D=\ell-1} q_{n_C,n_D|D} (n_C u_C(n_C,n_D) + n_D u_D(n_C,n_D)) \\ -(k-1) p_C z_C \sum_{n_C^{(2)}+n_D^{(2)}=\ell-1} q_{n_C,n_D|C} (n_C v_C(n_C,n_D) + n_D v_D(n_C,n_D)) \end{pmatrix} \right] \right\} + O\left(\theta^2\right) \tag{43}$$

Before plugging Eq. 22 into Eq. 43, we first derive useful identities to simplify Eq. 43. By the first $\ell - 1$ equations in Eq. 21, we have,

$$\sum_{m=1}^{\ell-1} (\ell - m)\, x_m = \frac{1}{k-1}, \tag{44}$$

and using this identity, we have

$$\begin{aligned} z_C &= \frac{(1-p_C)\,((\ell-1)\,k - \ell)}{k-1},\ \bar{z}_C := \sum_{n_C+n_D=\ell-1} n_C q_{(n_C,n_D)|C} = \ell - 1 - z_C, \\ z_D &= \frac{p_C\,((\ell-1)\,k - \ell)}{k-1},\ \bar{z}_D := \sum_{n_C+n_D=\ell-1} n_D q_{(n_C,n_D)|D} = \ell - 1 - z_D. \end{aligned} \tag{45}$$

We define

$$T_C := \sum_{n_C+n_D=\ell-1} q_{(n_C,n_D)|C} a_{n_C},\ T_D := \sum_{n_C+n_D=\ell-1} q_{(n_C,n_D)|D} b_{n_C}. \tag{46}$$

The payoffs in Eqs. 37-40 are simplified as

$$\begin{aligned} u_C(n_C, n_D) &= a_{n_C-1} + (k-1)T_C,\ v_C(n_C, n_D) = a_{n_C} + (k-1)T_C, \\ u_D(n_C, n_D) &= b_{n_C} + (k-1)T_D,\ v_D(n_C, n_D) = b_{n_C+1} + (k-1)T_D. \end{aligned} \tag{47}$$

And we also have the following identities

$$u_C(n_C+1, n_D-1) = v_C(n_C, n_D),\ u_D(n_C+1, n_D-1) = v_D(n_C, n_D). \tag{48}$$

Similar to Eq. 15, we can prove the following identities:

$$\begin{aligned}
p_D \sum_{n_C+n_D=\ell-1} n_C q_{(n_C,n_D)|D} u_C(n_C,n_D) &= p_C \sum_{n_C+n_D=\ell-1} n_D q_{(n_C,n_D)|C} v_C(n_C,n_D), \\
p_D \sum_{n_C+n_D=\ell-1} n_C q_{(n_C,n_D)|D} u_D(n_C,n_D) &= p_C \sum_{n_C+n_D=\ell-1} n_D q_{(n_C,n_D)|C} v_D(n_C,n_D), \\
p_D \sum_{n_C+n_D=\ell-1} n_C n_C q_{(n_C,n_D)|D} &= p_C \sum_{n_C+n_D=\ell-1} n_C n_D q_{(n_C,n_D)|C} + p_C z_C, \\
p_D \sum_{n_C+n_D=\ell-1} n_C n_D q_{(n_C,n_D)|D} &= p_C \sum_{n_C+n_D=\ell-1} n_D n_D q_{(n_C,n_D)|C} - p_C z_C, \\
p_D \sum_{n_C+n_D=\ell-1} n_C n_C q_{(n_C,n_D)|D} u_C(n_C,n_D) &= p_C \sum_{n_C+n_D=\ell-1} n_C n_D q_{(n_C,n_D)|C} v_C(n_C,n_D) \\
&\quad + p_C \sum_{n_C+n_D=\ell-1} n_D q_{(n_C,n_D)|C} v_C(n_C,n_D), \\
p_D \sum_{n_C+n_D=\ell-1} n_C n_D q_{(n_C,n_D)|D} u_D(n_C,n_D) &= p_C \sum_{n_C+n_D=\ell-1} n_D n_D q_{(n_C,n_D)|C} v_D(n_C,n_D) \\
&\quad - p_C \sum_{n_C+n_D=\ell-1} n_D q_{(n_C,n_D)|C} v_D(n_C,n_D), \\
p_D \sum_{n_C+n_D=\ell-1} n_D q_{(n_C,n_D)|D} u_D(n_C,n_D) &= k(\ell-1)(1-p_C)T_D - p_C \sum_{n_C+n_D=\ell-1} n_D q_{(n_C,n_D)|C} v_D(n_C,n_D), \\
p_C \sum_{n_C+n_D=\ell-1} n_C q_{(n_C,n_D)|C} v_C(n_C,n_D) &= k(\ell-1) p_C T_C - p_C \sum_{n_C+n_D=\ell-1} n_D q_{(n_C,n_D)|C} v_C(n_C,n_D).
\end{aligned} \tag{49}$$

Substituting Eq. 49 into Eq. 43 gives

$$\mathbb{E}\left(\Delta p_C\right) = \dot{p}_C = \frac{\theta}{N}\left(\frac{p_C \Omega}{k(\ell-1)} - \frac{k-1}{\ell-1} z_C p_C \left(T_D - T_C\right)\right) + O\left(\theta^2\right), \tag{50}$$

where

$$\begin{aligned}
\Omega &= \sum_{n_C+n_D=\ell-1} n_D \left(v_C^{(n_C,n_D)} - v_D^{(n_C,n_D)}\right) q_{(n_C,n_D)|C} \\
&= \sum_{m=0}^{\ell-1}\left(\sum_{n_C+n_D=\ell-1} n_D \left(a_{n_C} - b_{n_C+1}\right)\alpha_{n_C,n_D}^{(m)}\right) p_C^m + (k-1)\left(T_C - T_D\right) z_C
\end{aligned} . \tag{51}$$

In this case, we have

$$\begin{aligned}
\mu^{(m)} &:= \sum_{n_C+n_D=\ell-1, n_D \geq 1} n_D \left(a_{n_C} - b_{n_C+1}\right) \gamma_{n_C,n_D}^{(m)} \quad (0 \leq m \leq \ell-2),\ \mu^{(\ell-1)} := 0, \\
v^{(m)} &:= \sum_{n_C+n_D=\ell-1} b_{n_C}\beta_{n_C,n_D}^{(m)} - a_{n_C}\alpha_{n_C,n_D}^{(m)} \quad (0 \leq m \leq \ell-1), \\
\overline{\mu}^{(m)} &:= \frac{N(k-1)}{k\left[(\ell-1)k-\ell\right]}\mu^{(m)}, \\
\overline{v}^{(m)} &:= \frac{N(k+1)(k-1)}{k} v^{(m)}, \\
\overline{\tau}^{(m)} &:= \overline{\mu}^{(m)} - \overline{v}^{(m)}.
\end{aligned} \tag{52}$$

Plugging Eq. 52 into Eq. 34 gives the fixation probability of cooperation, and the condition for $\rho_C > 1/N$ (Eq. 35) becomes

$$\sum_{m=0}^{\ell-1} \frac{1}{(m+1)(m+2)}\left(\overline{\mu}^{(m)} - \overline{v}^{(m)}\right) > 0. \tag{53}$$

3.1 Well-mixed populations

In a well-mixed population, the average degree k equals $\binom{N-1}{\ell-1}$, which is much greater than 1 when $N \gg 1$. As a result, we only need to consider the terms of the highest order in k. We notice that the coefficients of $\overline{v}^{(m)}$ and $\overline{u}^{(m)}$ in Eq. 52 are $O(k)$ and $O(1/k)$. Then $\overline{u}^{(m)}$ in Eq. 53 is ignored, and Eq. 53 is equivalent to

$$\sum_{m=0}^{\ell-1} \frac{1}{(m+1)(m+2)} v^{(m)} < 0. \tag{54}$$

We then move to calculate the coefficient of $\alpha_{n_C,n_D}^{(m)}$ and $\beta_{n_C,n_D}^{(m)}$, keeping only the highest-order terms in k. By Eqs. 24-26, the coefficient of $\alpha_{n_C,n_D}^{(m)}$ is proportional to

$$\varphi_{n_C,n_D} = (-1)^{n_D} \binom{\ell-1}{n_D} \sum_{i=0}^{n_D} (-1)^{n_D-i} \binom{n_D}{i} \left(\frac{1}{i+n_C+1} - \frac{1}{i+n_C+2} \right). \tag{55}$$

Since

$$\begin{aligned} \sum_{i=0}^{\ell} \frac{1}{i+m} \binom{\ell}{i} (-1)^{l-i} &= \sum_{i=0}^{\ell} \left(\int_0^1 x^{i+m-1}\, dx \right) \binom{\ell}{i} (-1)^{\ell-i} = \int_0^1 x^{m-1} \left(\sum_{i=0}^{\ell} \binom{\ell}{i} (-1)^{\ell-i} x^i \right) dx \\ &= (-1)^{\ell} \int_0^1 x^{m-1} (1-x)^{\ell}\, dx = (-1)^{\ell} \frac{(m-1)!\ell!}{(m+\ell)!}, \end{aligned} \tag{56}$$

we have

$$\varphi_{n_C,n_D} = \binom{\ell-1}{n_D} \left(\frac{n_C! n_D!}{\ell!} - \frac{(n_C+1)! n_D!}{(\ell+1)!} \right) = \frac{n_D+1}{\ell(\ell+1)}. \tag{57}$$

Similarly, we can prove that the coefficient of $\beta_{n_C,n_D}^{(m)}$ is also proportional to $(n_D+1)/(\ell(\ell+1))$. Then, $\rho_C > 1/N$ holds when

$$\sum_{n_C=0}^{\ell-1} (\ell-n_C) a_{n_C} > \sum_{n_C=0}^{\ell-1} (\ell-n_C) b_{n_C}, \tag{58}$$

which recovers the results in Ref. (2)

3.2 k-regular graphs

In this section, we provide explicit results for $\ell = 2$ and $\ell = 3$ on hypergraphs of average degree k. For $\ell = 2$, we have

$$\begin{aligned} &\mu^{(0)} = (a_0 - b_1)\, \alpha_{0,1}^{(0)},\ \mu^{(1)} = 0,\ v^{(m)} = b_0 \beta_{0,1}^{(m)} + b_1 \beta_{1,0}^{(m)} - a_0 \alpha_{0,1}^{(m)} - a_1 \alpha_{1,0}^{(m)} \quad (m = 0, 1), \\ &\overline{\mu}^{(m)} = \frac{N(k-1)}{k(k-2)} \mu^{(m)},\ \overline{v}^{(m)} = \frac{N(k+1)(k-1)}{k} v^{(m)}, \\ &\alpha_{1,0}^{(0)} = \frac{1}{k-1},\ \alpha_{1,0}^{(1)} = \frac{k-2}{k-1},\ \alpha_{0,1}^{(0)} = \frac{k-2}{k-1},\ \alpha_{0,1}^{(1)} = -\frac{k-2}{k-1}, \\ &\beta_{1,0}^{(0)} = 0,\ \beta_{1,0}^{(1)} = \frac{k-2}{k-1},\ \beta_{0,1}^{(0)} = 1,\ \beta_{0,1}^{(1)} = -\frac{k-2}{k-1}. \end{aligned} \tag{59}$$

Plugging Eq. 59 into Eq. 53, we have

$$(2k^2 - 2k - 1) a_0 + (k^2 + 2k + 1) a_1 > (2k^2 + k - 1) b_0 + (k^2 - k + 1) b_1, \tag{60}$$

which recovers the result in pairwise regular graphs, derived in Ref. (1).

For $\ell = 3$, we have

$$
\begin{aligned}
&\mu^{(0)} = (a_1 - b_2)\,\gamma^{(0)}_{1,1} + 2\,(a_0 - b_1)\,\gamma^{(0)}_{0,2},\ \mu^{(1)} = (a_1 - b_2)\,\gamma^{(1)}_{1,1} + 2\,(a_0 - b_1)\,\gamma^{(1)}_{0,2},\ \mu^{(2)} = 0,\\
&\gamma^{(0)}_{1,1} = \alpha^{(0)}_{1,1},\ \gamma^{(0)}_{0,2} = \alpha^{(0)}_{0,2},\ \gamma^{(1)}_{1,1} = \alpha^{(1)}_{1,1} + \alpha^{(0)}_{1,1},\ \gamma^{(1)}_{0,2} = \alpha^{(1)}_{0,2} + \alpha^{(0)}_{0,2},\\
&v^{(m)} = b_0\beta^{(m)}_{0,2} + b_1\beta^{(m)}_{1,1} + b_2\beta^{(m)}_{2,0} - a_0\alpha^{(m)}_{0,2} - a_1\alpha^{(m)}_{1,1} - a_2\alpha^{(m)}_{2,0} \quad (m = 0,1,2),\\
&\overline{\mu}^{(m)} = \frac{N(k-1)}{k(2k-3)}\mu^{(m)},\ \overline{v}^{(m)} = \frac{N(k+1)(k-1)}{k}v^{(m)},
\end{aligned}
\tag{61}
$$

and

$$
\begin{aligned}
&\alpha^{(0)}_{2,0} = \frac{1}{(2k-1)(k-1)},\ \alpha^{(1)}_{2,0} = \frac{6k-9}{2(2k-1)(k-1)},\ \alpha^{(2)}_{2,0} = \frac{(2k-3)^2}{2(2k-1)(k-1)},\\
&\alpha^{(0)}_{1,1} = \frac{2k-3}{(2k-1)(k-1)},\ \alpha^{(1)}_{1,1} = \frac{(2k-3)(2k-4)}{(2k-1)(k-1)},\ \alpha^{(2)}_{1,1} = -\frac{(2k-3)^2}{(2k-1)(k-1)},\\
&\alpha^{(0)}_{0,2} = \frac{(k-1)(2k-3)}{(2k-1)(k-1)},\ \alpha^{(1)}_{0,2} = -\frac{(2k-3)(4k-5)}{2(2k-1)(k-1)},\ \alpha^{(2)}_{0,2} = \frac{(2k-3)^2}{2(2k-1)(k-1)},\\
&\beta^{(0)}_{2,0} = 0,\ \beta^{(1)}_{2,0} = \frac{2k-3}{2(2k-1)(k-1)},\ \beta^{(2)}_{2,0} = \frac{(2k-3)^2}{2(2k-1)(k-1)},\\
&\beta^{(0)}_{1,1} = 0,\ \beta^{(1)}_{1,1} = \frac{(2k-2)(2k-3)}{(2k-1)(k-1)},\ \beta^{(2)}_{1,1} = -\frac{(2k-3)^2}{(2k-1)(k-1)},\\
&\beta^{(0)}_{0,2} = 1,\ \beta^{(1)}_{0,2} = -\frac{(2k-3)(4k-3)}{2(2k-1)(k-1)},\ \beta^{(2)}_{0,2} = \frac{(2k-3)^2}{2(2k-1)(k-1)}.
\end{aligned}
\tag{62}
$$

In this case, $\rho_C > 1/N$ is equivalent to

$$
\begin{aligned}
&(12k^3 - 16k^2 + 3k + 3)a_0 + (8k^3 - 6k - 6)a_1 + (4k^3 + 4k^2 + 3k + 3)a_2\\
&> (12k^3 - 9k + 3)b_0 + (8k^3 - 8k^2 + 6k - 6)b_1 + (4k^3 - 4k^2 + 3k + 3)b_2
\end{aligned}.
\tag{63}
$$

We then consider two specific types of multiplayer social dilemma: the donation game and the nonlinear public goods game.

Donation game (DG). The two-player donation game has the following payoff matrix:

$$
\begin{pmatrix} a_0 & a_1 \\ b_0 & b_1 \end{pmatrix} = \begin{pmatrix} -c & -c+b \\ 0 & b \end{pmatrix}.
\tag{64}
$$

Plugging these elements into Eq. 60 recovers the well-known $b/c > k$ condition for cooperation to be favored.

To investigate the structure of the multi-player donation game, which leads to the $b/c > k$ condition, we begin by analyzing the case of $\ell = 3$ with Eq. 63. We notice that when the three-player game has the following payoff matrix:

$$
\begin{pmatrix} a_0 & a_1 & a_2 \\ b_0 & b_1 & b_2 \end{pmatrix} = \begin{pmatrix} -c & -c+b & -c+2b \\ 0 & b & 2b \end{pmatrix},
\tag{65}
$$

Eq. 63 is equivalent to $b/c > k$.

Based on this observation, we turn to prove that, for any ℓ, the $b/c > k$ condition holds when

$$
\begin{pmatrix} a_0 & a_1 & \dots & a_{\ell-1} \\ b_0 & b_1 & \dots & b_{\ell-1} \end{pmatrix} = \begin{pmatrix} -c & -c+b & \dots & -c+(\ell-1)b \\ 0 & b & \dots & (\ell-1)b \end{pmatrix}.
\tag{66}
$$

In this case, instead of calculating every $\alpha^{(m)}_{n_C,n_D}$ and $\beta^{(m)}_{n_C,n_D}$, we directly calculate the quantities Ω, T_C, and T_D. We

have

$$\begin{aligned}
T_C &= -c + b\left(\ell - 1 - \frac{(1-p_C)\left((\ell-1)k-\ell\right)}{k-1}\right), \\
T_D &= b\frac{p_C\left((\ell-1)k-\ell\right)}{k-1}, \\
\Omega &= \frac{(1-p_C)\left((\ell-1)k-\ell\right)}{k-1}\left((k-1)(T_C-T_D)-(b+c)\right).
\end{aligned} \tag{67}$$

Plugging these quantities into Eq. 32 gives

$$\frac{2\mathbb{E}(\Delta p_C)}{\mathrm{Var}(\Delta p_C)} = \theta N\,(b-kc)\,, \tag{68}$$

which means

$$\overline{\tau}^{(0)} = N\,(b-kc)\,,\ \overline{\tau}^{(m)} = 0 \quad (1 \le m \le \ell - 1)\,. \tag{69}$$

Then, we obtain the $b/c > k$ condition by Eq. 53.

Public goods game (PGG). For the two-player public goods game

$$\begin{pmatrix} a_0 & a_1 \\ b_0 & b_1 \end{pmatrix} = \begin{pmatrix} -c+\dfrac{b}{2} & -c+\dfrac{(\delta_2+1)b}{2} \\ 0 & \dfrac{b}{2} \end{pmatrix}, \tag{70}$$

and the three-player public goods game

$$\begin{pmatrix} a_0 & a_1 & a_2 \\ b_0 & b_1 & b_2 \end{pmatrix} = \begin{pmatrix} -c+\dfrac{b}{3} & -c+\dfrac{(\delta_3+1)b}{3} & -c+\dfrac{(\delta_3^2+\delta_3+1)b}{3} \\ 0 & \dfrac{b}{3} & \dfrac{(\delta_3+1)b}{3} \end{pmatrix}, \tag{71}$$

we have

$$\rho_C(1/N) > \frac{1}{N} \Leftrightarrow \frac{b}{c} > \left(\frac{b}{c}\right)^* = \begin{cases} \dfrac{6k^2}{(k+1)(k(\delta_2+2)+\delta_2-1)}, & \ell = 2, \\ \dfrac{36k^2(2k-1)}{(k+1)\left(\delta_3^2\left(4k^2+3\right)+\delta_3\left(8k^2-6\right)+3(2k-1)^2\right)}, & \ell = 3. \end{cases} \tag{72}$$

4 Neutral complex contagion

The complexity of higher-order interactions in evolutionary dynamics arises from game interactions. In this section, we move to analyze neutral complex contagion on hypergraphs, where this complexity is at work during the strategy updating process. In this case, strategies C and D can be interpreted as two competing opinions. The term "neutral" here indicates that neither opinion has an evolutionary advantage over the other. More specifically, we have

$$\begin{aligned}
F_C(n_C, n_D; \theta) &= G_C(n_C, n_D; \theta) = n_C^{1+\lambda\theta}, \\
F_D(n_C, n_D; \theta) &= G_D(n_C, n_D; \theta) = n_D^{1+\lambda\theta},
\end{aligned} \tag{73}$$

where λ represents the complexity parameter in contagion dynamics. When $\lambda = 0$, opinions spread via simple contagion. Otherwise, opinions spread via complex contagion. We have

$$\begin{aligned}
\partial_\theta F_C(n_C, n_D; 0) &= \partial_\theta G_C(n_C, n_D; 0) = \lambda n_C \ln n_C, \\
\partial_\theta F_D(n_C, n_D; 0) &= \partial_\theta G_D(n_C, n_D; 0) = \lambda n_D \ln n_D.
\end{aligned} \tag{74}$$

Similar to Eq. 49, in this case, we can prove the following identities

$$
\begin{aligned}
p_D \sum_{n_C+n_D=\ell-1} q_{(n_C,n_D)|D} n_C \ln n_C &= p_C \sum_{n_C+n_D=\ell-1} q_{(n_C,n_D)|C} n_D \ln(n_C+1), \\
p_D \sum_{n_C+n_D=\ell-1} q_{(n_C,n_D)|D} n_C n_C \ln n_C &= p_C \sum_{n_C+n_D=\ell-1} q_{(n_C,n_D)|C} n_D n_C \ln(n_C+1) \\
&\quad + p_C \sum_{n_C+n_D=\ell-1} q_{(n_C,n_D)|C} n_D \ln(n_C+1), \\
p_D \sum_{n_C+n_D=\ell-1} q_{(n_C,n_D)|D} n_C n_D \ln n_D &= p_C \sum_{n_C+n_D=\ell-1, n_D\geq 2} q_{(n_C,n_D)|C} n_D n_D \ln(n_D-1) \\
&\quad - p_C \sum_{n_C+n_D=\ell-1, n_D\geq 2} q_{(n_C,n_D)|C} n_D \ln(n_D-1).
\end{aligned} \tag{75}
$$

As a result, the time derivative of p_C becomes

$$
\begin{aligned}
\dot{p}_C = \mathbb{E}(\Delta p_C) = \frac{\lambda\theta}{N} \Bigg\{ & \frac{p_C \sum_{n_C+n_D=\ell-1} q_{(n_C,n_D)|C} n_D \ln \frac{n_C+1}{n_D}}{\ell-1} - \frac{1}{k(\ell-1)^2} \Bigg[\begin{pmatrix} p_C \sum_{n_C+n_D=\ell-1} q_{(n_C,n_D)|C} n_C n_D \ln\left(\frac{n_C+1}{n_C}\right) \\ +p_C \sum_{n_C+n_D=\ell-1} q_{(n_C,n_D)|C} n_D \ln(n_C+1) \end{pmatrix} \\
& + \begin{pmatrix} p_C \sum_{n_C+n_D=\ell-1, n_D\geq 2} q_{(n_C,n_D)|C} n_D n_D \ln(n_D-1) \\ -p_C \sum_{n_C+n_D=\ell-1, n_D\geq 2} q_{(n_C,n_D)|C} n_D \ln(n_D-1) \\ -p_C \sum_{n_C+n_D=\ell-1} q_{(n_C,n_D)|C} n_D n_D \ln n_D \end{pmatrix} + \begin{pmatrix} (k-1) p_D z_D \sum_{n_C+n_D=\ell-1} q_{(n_C,n_D)|D} (n_C \ln n_C + n_D \ln n_D) \\ -(k-1) p_C z_C \sum_{n_C+n_D=\ell-1} q_{(n_C,n_D)|C} (n_C \ln n_C + n_D \ln n_D) \end{pmatrix} \Bigg] \Bigg\} + O\left(\theta^2\right).
\end{aligned} \tag{76}
$$

The variance of Δp_C remains identical to Eq. 28.

We define

$$
\begin{aligned}
\mu^{(m)} &:= \sum_{n_C+n_D=\ell-1} n_D \ln \frac{n_C+1}{n_D} \gamma^{(m)}_{n_C,n_D} \quad (0 \leq m \leq \ell-2),\ \mu^{(\ell-1)} := 0, \\
\xi^{(m)}_1 &:= \sum_{n_C+n_D=\ell-1} n_C n_D \ln \frac{n_C+1}{n_C} \gamma^{(m)}_{n_C,n_D} \quad (0 \leq m \leq \ell-2),\ \xi^{(\ell-1)}_1 := 0, \\
\xi^{(m)}_2 &:= \sum_{n_C+n_D=\ell-1} n_D \ln(n_C+1) \gamma^{(m)}_{n_C,n_D} \quad (0 \leq m \leq \ell-2),\ \xi^{(\ell-1)}_2 := 0, \\
\xi^{(m)}_3 &:= \sum_{n_C+n_D=\ell-1} n_D n_D \ln n_D \gamma^{(m)}_{n_C,n_D} \quad (0 \leq m \leq \ell-2),\ \xi^{(\ell-1)}_3 := 0, \\
\xi^{(m)}_4 &:= \sum_{n_C+n_D=\ell-1, n_D\geq 2} n_D n_D \ln(n_D-1) \gamma^{(m)}_{n_C,n_D} \quad (0 \leq m \leq \ell-2),\ \xi^{(\ell-1)}_4 := 0, \\
\xi^{(m)}_5 &:= \sum_{n_C+n_D=\ell-1, n_D\geq 2} n_D \ln(n_D-1) \gamma^{(m)}_{n_C,n_D} \quad (0 \leq m \leq \ell-2),\ \xi^{(\ell-1)}_5 := 0, \\
v^{(m)} &:= \sum_{n_C+n_D=\ell-1} (n_C \ln n_C + n_D \ln n_D) \left(\beta^{(m)}_{n_C,n_D} - \alpha^{(m)}_{n_C,n_D}\right), \\
\overline{\mu}^{(m)} &:= \frac{\lambda N(k-1)}{(\ell-1)k-\ell} \mu^{(m)}, \\
\overline{\xi}^{(m)}_i &:= \frac{\lambda N(k-1)}{k(\ell-1)\left[(\ell-1)k-\ell\right]} \xi^{(m)}_i \quad (i=1,2,3,4,5), \\
\overline{v}^{(m)} &:= \frac{\lambda N(k-1)}{k(\ell-1)} v^{(m)}, \\
\overline{\tau}^{(m)} &:= \overline{\mu}^{(m)} - \overline{\xi}^{(m)}_1 - \overline{\xi}^{(m)}_2 + \overline{\xi}^{(m)}_3 - \overline{\xi}^{(m)}_4 + \overline{\xi}^{(m)}_5 - \overline{v}^{(m)}.
\end{aligned} \tag{77}
$$

In addition, to ensure these definitions are all well-defined, when $n_C = 0$, we set $n_C \ln n_C = \lim_{n_C\to 0} n_C \ln n_C = 0$ and $n_C \ln(1/n_C) = \lim_{n_C\to 0} n_C \ln(1/n_C) = 0$. Plugging Eq. 77 into Eq. 34 gives the fixation probability in the regime of neutral complex contagion.

For pairwise graphs (i.e. $\ell = 2$), there is no complexity in contagion dynamics, so the evolutionary process behaves as a martingale, and the fixation probability of opinion C is equal to its initial frequency p_0. However,

when the contagion occurs on hypergraphs, strategy C spreads via complex contagion. As an illustrative example, we calculate the fixation probability of C when $\ell = 3$. We have

$$
\begin{aligned}
&\mu^{(0)} = \ln 2 \cdot \gamma_{1,1}^{(0)} - 2\ln 2 \cdot \gamma_{0,2}^{(0)},\ \mu^{(1)} = \ln 2 \cdot \gamma_{1,1}^{(1)} - 2\ln 2 \cdot \gamma_{0,2}^{(1)},\ \mu^{(2)} = 0,\\
&\xi_1^{(0)} = \xi_2^{(0)} = \ln 2 \cdot \gamma_{1,1}^{(0)},\ \xi_1^{(1)} = \xi_2^{(1)} = \ln 2 \cdot \gamma_{1,1}^{(1)}, \xi_1^{(2)} = \xi_2^{(2)} = 0,\\
&\xi_3^{(0)} = 4\ln 2 \cdot \gamma_{0,2}^{(0)},\ \xi_3^{(1)} = 4\ln 2 \gamma_{0,2}^{(1)},\ \xi_3^{(2)} = 0,\\
&\gamma_{1,1}^{(0)} = \alpha_{1,1}^{(0)},\ \gamma_{0,2}^{(0)} = \alpha_{0,2}^{(0)},\ \gamma_{1,1}^{(1)} = \alpha_{1,1}^{(0)} + \alpha_{1,1}^{(1)},\ \gamma_{0,2}^{(1)} = \alpha_{0,2}^{(0)} + \alpha_{0,2}^{(1)},\\
&\varphi_1^{(m)} = \varphi_2^{(m)} = 0 \quad (m = 0, 1, 2),\\
&v^{(m)} = 2\ln 2 \left(\beta_{0,2}^{(m)} + \beta_{2,0}^{(m)} - \alpha_{0,2}^{(m)} - \alpha_{2,0}^{(m)}\right) \quad (m = 0, 1, 2),\\
&\overline{\mu}^{(m)} = \frac{\lambda N(k-1)}{2k-3}\mu^{(m)},\ \overline{\xi}_i^{(m)} = \frac{\lambda N(k-1)}{2k(2k-3)}\xi_i^{(m)},\ \overline{v}^{(m)} = \frac{\lambda N(k-1)}{2k}v^{(m)}.
\end{aligned}
\tag{78}
$$

In this case,

$$
\rho(p_0) = p_0 + \theta N \lambda \ln 2 \cdot \frac{2k-3}{6(2k-1)} p_0(1-p_0)(2p_0-1) + O\left(\theta^2\right). \tag{79}
$$

and

$$
\rho_C = \frac{1}{N} + \theta\lambda \ln 2 \cdot \frac{2k-3}{2k-1}\left(-\frac{1}{6} + \frac{1}{2N} - \frac{1}{3N^2}\right) + O\left(\theta^2\right). \tag{80}
$$

In the large population limit $N \to \infty$,

$$
\rho_C = \frac{1}{N} - \frac{\theta\lambda \ln 2 \cdot (2k-3)}{6(2k-1)} + O\left(\theta^2\right). \tag{81}
$$

Since the contagion dynamics are symmetric with respect to both strategies, the fixation probability of D, ρ_D, is identical to ρ_C. In well-mixed populations, the first-order term of ρ_C approaches $-\lambda \ln 2/6$.

5 Complex contagion with selection

In evolutionary game dynamics, higher-order interactions govern an individual's fitness and strategic updates, but the strategy updating process remains pairwise-based: each individual copies the strategy of another individual. Conversely, in a neutral complex contagion, all individuals have the same fitness, but the strategy updating process is governed by higher-order interactions: an individual inherits a strategy from its interaction neighborhood rather than from another individual. Therefore, a straightforward extension is to simultaneously consider higher-order interactions in both the assignment of fitness and in the process of strategic updating.

We analyze the following combination:

$$
\begin{aligned}
F_C(n_C, n_D; \theta) = n_C^{1+\lambda\theta} f_C(n_C, n_D),\ G_C(n_C, n_D; \theta) = n_C^{1+\lambda\theta} g_C(n_C, n_D),\\
F_D(n_C, n_D; \theta) = n_D^{1+\lambda\theta} f_D(n_C, n_D), G_D(n_C, n_D; \theta) = n_D^{1+\lambda\theta} g_D(n_C, n_D).
\end{aligned}
\tag{82}
$$

The derivatives with respect to θ at $\theta = 0$ are

$$
\begin{aligned}
\partial_\theta F_C(n_C, n_D; 0) = n_C u_C(n_C, n_D) + \lambda n_C \ln n_C,\ \partial_\theta G_C(n_C, n_D; 0) = n_C v_C(n_C, n_D) + \lambda n_C \ln n_C,\\
\partial_\theta F_D(n_C, n_D; 0) = n_D u_D(n_C, n_D) + \lambda n_D \ln n_D,\ \partial_\theta G_D(n_C, n_D; 0) = n_D v_D(n_C, n_D) + \lambda n_D \ln n_D,
\end{aligned}
\tag{83}
$$

which are the sum of the derivatives in the cases of evolutionary game dynamics (Eq. 42) and complex contagion (Eq. 74). Then the expectation $\mathbb{E}(\Delta p_C)$ in this case is also the sum of Eq. 50 and Eq. 76.

We are interested in how complex contagion affects the evolution of cooperation on hypergraphs. Specifically, we analyze the effect of the complexity parameter λ on the critical benefit-to-cost ratio $(b/c)^*$. We first derive the

fixation probability ρ_C under a general three-player game. For a large population size N, we have

$$\rho_C = \frac{1}{N} + \frac{\theta}{24k(2k-1)} \left(\substack{a_0(12k^3-16k^2+3k+3)+a_1(8k^3-6k-6)+a_2(4k^3+4k^2+3k+3) \\ -b_0(12k^3-9k+3)-b_1(8k^3-8k^2+6k-6)-b_2(4k^3-4k^2+3k+3)} - 4\lambda k \cdot \ln 2\,(2k-3) \right) + O\left(\theta^2\right). \tag{84}$$

For the three-player donation game (Eq. 65), the fixation probability is

$$\rho_C = \frac{1}{N} + \frac{\theta}{24(2k-1)} \left(12(2k-1)(b-ck) - 4\lambda \cdot \ln 2(2k-3)\right) + O\left(\theta^2\right). \tag{85}$$

For the three-player public goods game (Eq. 71), the fixation probability is

$$\rho_C = \frac{1}{N} + \frac{\theta}{72k(2k-1)} \left(\substack{(k+1)\left(\delta_3^2(4k^2+3)+\delta_3(8k^2-6)+3(2k-1)^2\right) \\ -3k(12k(2k-1)c+4\lambda\cdot\ln 2(2k-3))} \right) + O\left(\theta^2\right). \tag{86}$$

When $c = 1$, the critical benefit-to-cost ratio in these two games becomes

$$\left(\frac{b}{c}\right)^* = \begin{cases} k + \dfrac{\lambda \ln 2 \cdot (2k-3)}{3(2k-1)}, & \text{Donation game,} \\ \dfrac{36k^2(2k-1) + 12\lambda k \cdot \ln 2(2k-3)}{(k+1)\left(\delta_3^2\left(4k^2+3\right) + \delta_3\left(8k^2-6\right) + 3(2k-1)^2\right)}, & \text{Public goods game.} \end{cases} \tag{87}$$

When the evolutionary game dynamics are driven by the average payoffs (i.e., u and v are replaced by u/k and v/k), the critical ratios become

$$\left(\frac{b}{c}\right)^* = \begin{cases} k + \dfrac{\lambda k \ln 2 \cdot (2k-3)}{3(2k-1)}, & \text{Donation game,} \\ \dfrac{36k^2(2k-1) + 12\lambda k^2 \cdot \ln 2(2k-3)}{(k+1)\left(\delta_3^2\left(4k^2+3\right) + \delta_3\left(8k^2-6\right) + 3(2k-1)^2\right)}, & \text{Public goods game.} \end{cases} \tag{88}$$

6 Generation of random hypergraphs

In this work, beyond the regular hypergraph considered in the main text, we also investigate the performance of diffusion approximation on random hypergraphs with pure third-order interactions, including both random regular hypergraphs and heterogeneous hypergraphs generated by the preferential attachment rule, which was introduced in the Barabási-Albert model (3).

A random regular hypergraph with third-order interactions is generated by the configuration model outlined in Algorithm 1. The basic idea is to assign each node k stubs and then randomly shuffle and group these stubs into triples, each forming a hyperedge. The same idea can be extended to generate ℓth-order random regular graphs.

Algorithm 1 Configuration model for random regular hypergraphs with third-order interactions

Input: Number of nodes N, average degree k, maximum retries T
Output: Set of hyperedges

Check if $3 \mid Nk$. If not, return "No valid hypergraph"
Set $M \leftarrow Nk/3$: the total number of hyperedges
for $t = 1$ to T **do**
    Randomly permute stubs
    Partition stubs into M groups of size 3
    Let each group be a candidate hyperedge
    if every hyperedge has 3 distinct vertices **then**
        if no duplicate hyperedges **then**
            Count vertex occurrences across hyperedges
            if each vertex appears exactly k times and the graph is connected **then return** Set of hyperedges
return "Construction failed, try again"

For heterogeneous hypergraphs, the construction begins with a complete graph of size m_0, that is, the initial hyperedges consist of all possible 3-node combinations. Then, at each time step, add a node and create m new hyperedges that connect the new node to two existing nodes chosen preferentially by hyperdegree. The construction process stops after T time steps. Algorithm 2 outlines the construction procedure, which can also be extended to hypergraphs of arbitrary order ℓ.

Algorithm 2 Preferential attachment hypergraph model ($\ell = 3$)

Input: Number of time steps T, initial graph size m_0, linking number m
Output: Set of hyperedges

Initialize hypergraph with m_0 nodes
Form all possible 3-node hyperedges among the m_0 nodes
Set the hyperdegree of each seed node equal to $\binom{m_0-1}{2}$
for $t = 1$ to T **do**
    Add a new node v with hyperdegree 0
    for $j = 1$ to m **do**
        Select an existing node u with probability proportional to its hyperdegree
        Select another distinct node w with probability proportional to its hyperdegree
        Form a new hyperedge (v, u, w)
        Increase hyperdegree of v, u, w by 1
return Set of hyperedges

References

[1] H. Ohtsuki, C. Hauert, E. Lieberman, and M. A. Nowak. A simple rule for the evolution of cooperation on graphs and social networks. *Nature*, 441(7092):502–505, 2006.

[2] C. S. Gokhale and A. Traulsen. Evolutionary games in the multiverse. *Proceedings of the National Academy of Sciences*, 107(12):5500–5504, 2010.

[3] A.-L. Barabási and R. Albert. Emergence of scaling in random networks. *Science*, 286(5439):509–512, 1999.

[4] A. Sheng, Q. Su, L. Wang, and J. B. Plotkin. Strategy evolution on higher-order networks. *Nature Computational Science*, 4:274–284, 2024.

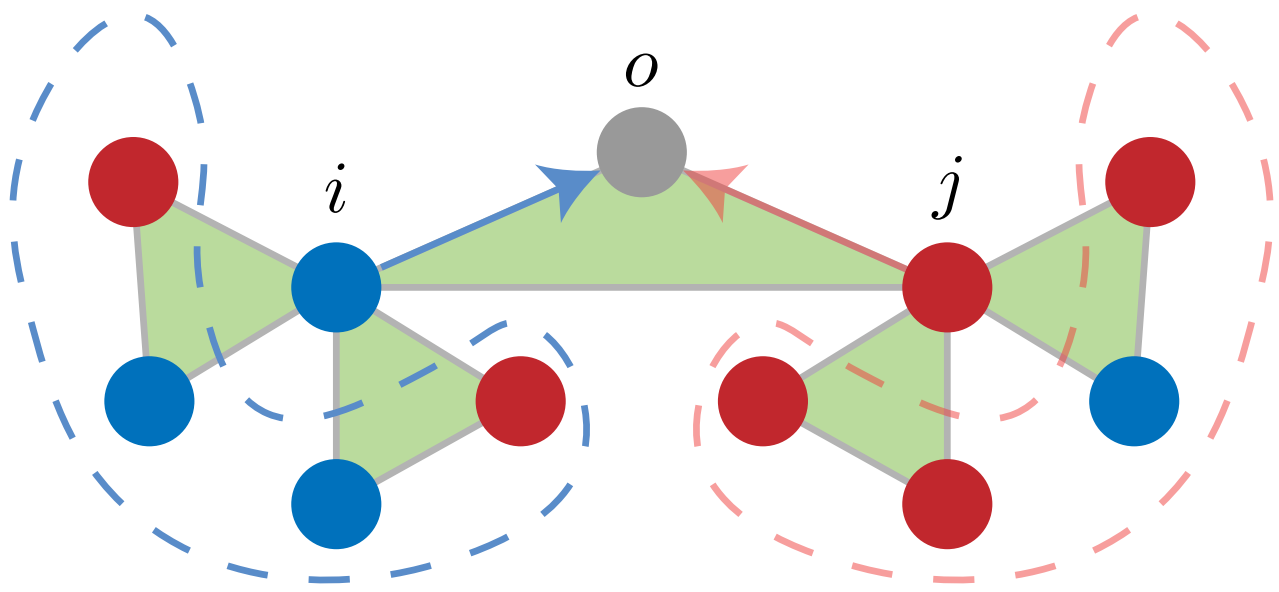

$$N_{C|C} = \sum_{n_C+n_D=\ell-1} n_C q_{(n_C,n_D)|C} \quad u_i = (k-1)N_{C|C}b - ck$$

$$N_{C|D} = \sum_{n_C+n_D=\ell-1} n_C q_{(n_C,n_D)|D} \quad u_j = (k-1)N_{C|D}b$$

$$(k-1)\left(N_{C|C} - N_{C|D}\right) = 1$$

$$u_i > u_j \Leftrightarrow \frac{b}{c} > k$$

Figure S1: Intuition for the multiplayer donation game on hypergraphs. As an illustrative example, we consider the intuition behind the $b/c > k$ rule on hypergraphs of order $\ell = 3$. Suppose an individual o is selected to be replaced, and a cooperator i and a defector j compete for this site. Let $N_{C|C}$ and $N_{C|D}$ denote the average number of cooperative neighbors of a focal cooperation and a focal defector per interaction, respectively. Individual i pays cost ck and receives benefit $(k-1)N_{C|C}b$ from the remaining $(k-1)$ higher-order interactions, while individual j pays no cost but still receives benefit $(k-1)N_{C|D}b$ from cooperative neighbors. Selection favors cooperation when the payoff of i, u_i, is larger than that of j, u_j. Since a cooperator has one more cooperative neighbor than a defector on average (Eq. 44), this condition becomes the $b/c > k$ rule.

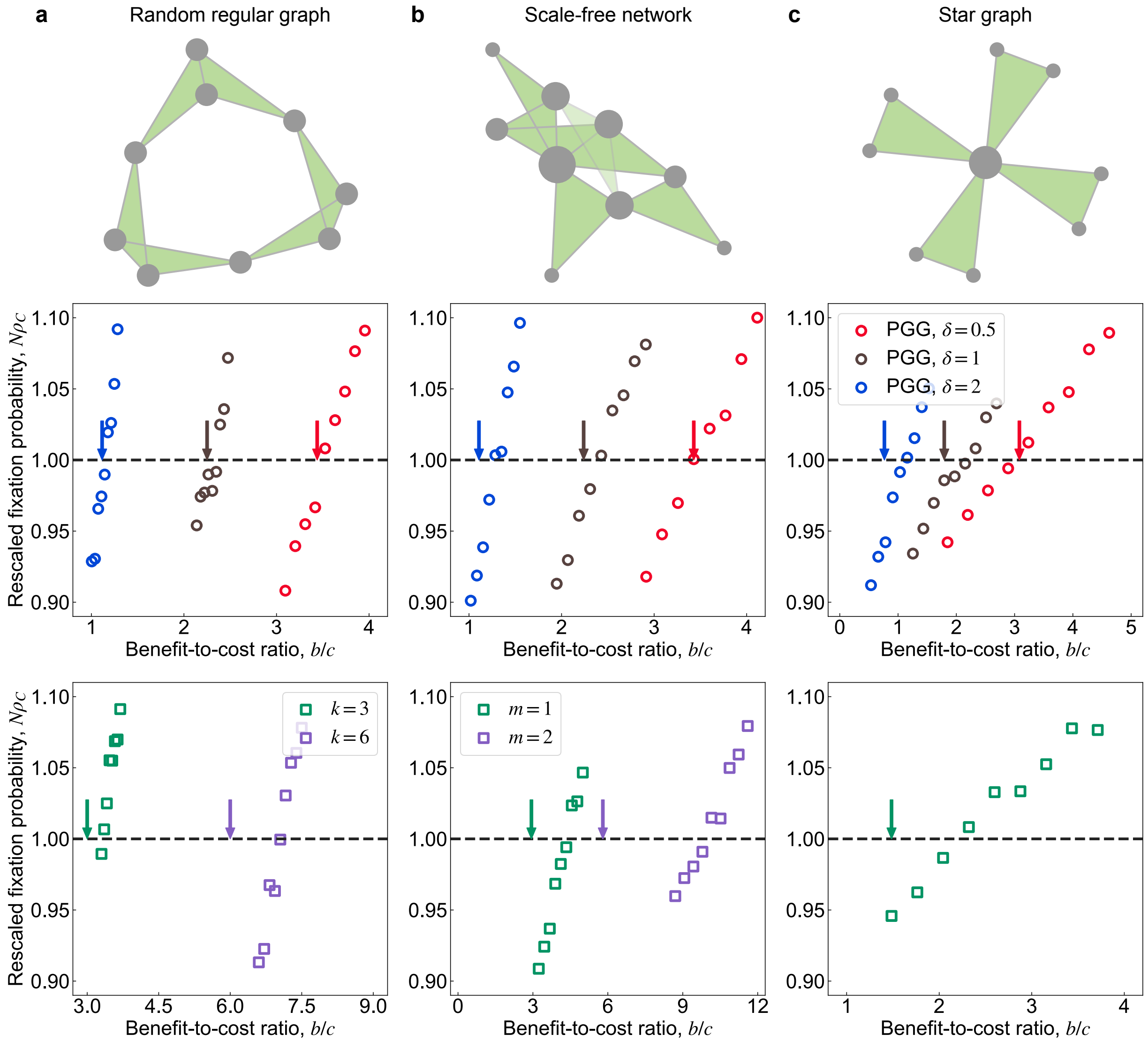

Figure S2: Evolutionary game dynamics on random hypergraphs. We consider three types of random hypergraphs: random regular hypergraphs with average degree k (**a**) generated by the configuration model (see Algorithm 1); scale-free hypergraphs with linking number m (**b**) generated by the preferential attachment model (see Algorithm 2); higher-order star graphs (**c**) (4) . We calculate the fixation probability of cooperation under the public goods game (the middle panels) and the donation game (the bottom panels), by averaging over 2×10^6 independent Monte Carlo simulations. Arrows show the theoretical predictions. Although structural heterogeneity causes discrepancies between theory and simulations, the critical benefit-to-cost ratio derived by the ℓ-hyperedge approximation still provides a necessary condition for the evolution of cooperation. Parameters: $c = 1$, $\theta = 0.01$, $k = 3$ and $m = 1$ in the public goods game.

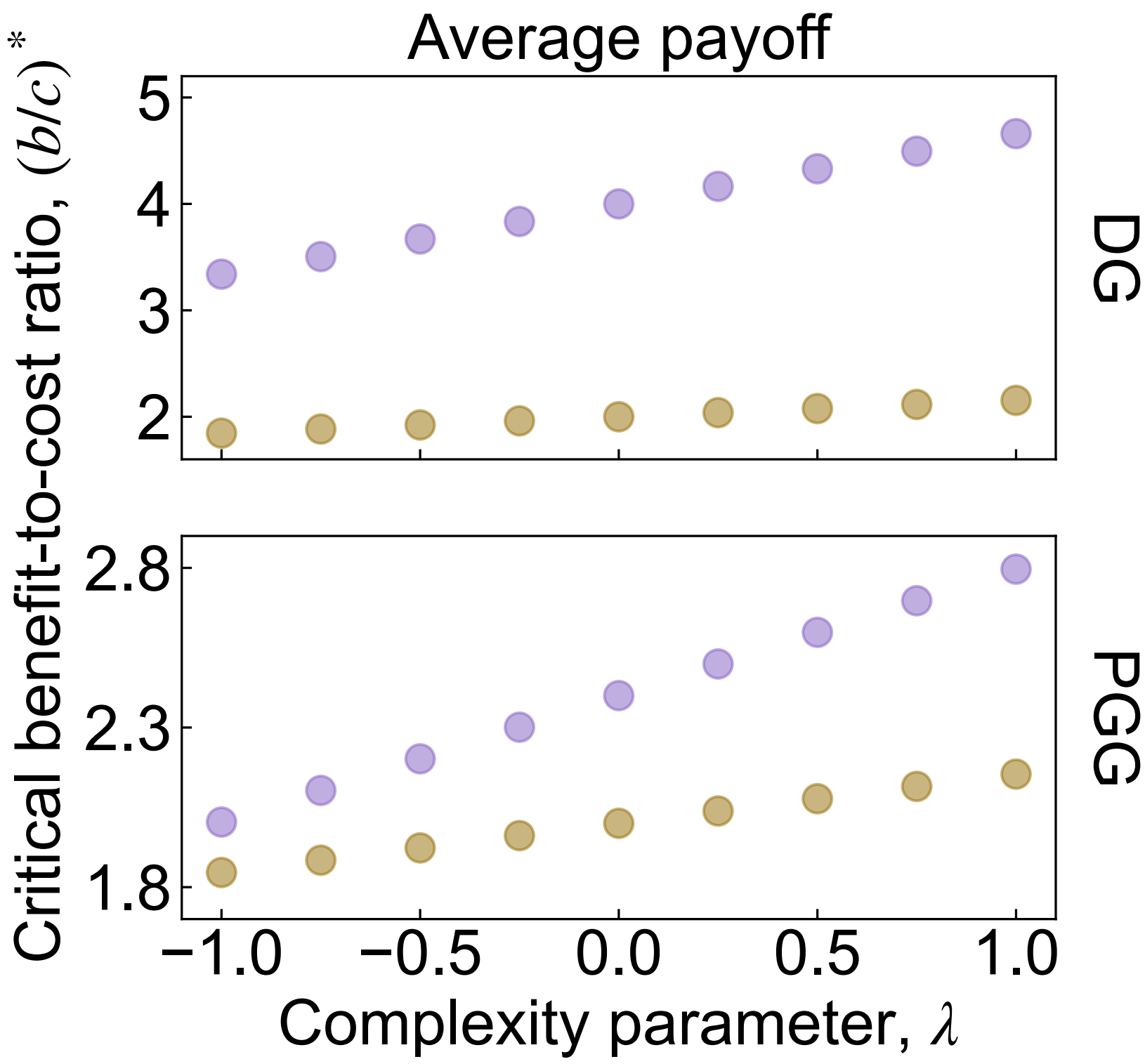

Figure S3: Critical benefit-to-cost ratio with complex contagion under the average-payoff setting. The effects of the game and complex contagion on the fixation probability ρ_C have an identical order in k, then the variants in the critical benefit-to-cost ratio $(b/c)^*$ are more significant as λ changes, compared to that under the accumulated-payoff setting.

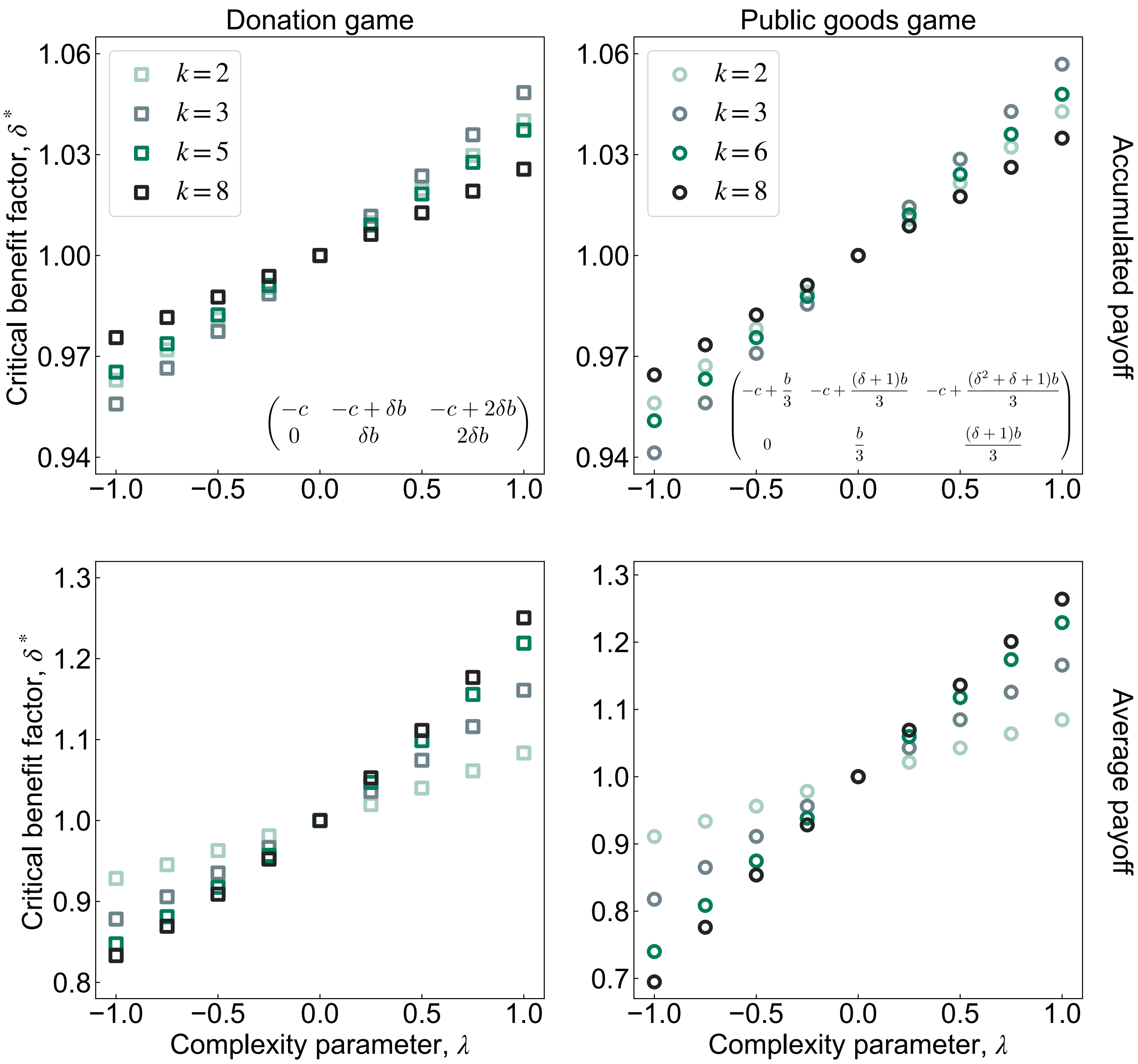

Figure S4: Critical benefit factor for cooperation under complex contagion. We calculate the critical benefit factor δ^* that maintains the same cooperation threshold $(b/c)^*$ as in the case without complex contagion ($\lambda = 0$). The critical benefit factor δ^* increases monotonically with λ. Similar to the results in Fig. 4c and Fig. S3, this trend is more significant under the average-payoff setting.
